# Soil-Easily Extractable Glomalin: An Innovative Approach to Deciphering Its Molecular Composition under the Influence of Seasonality, Vegetation Cover, and Wildfire

**DOI:** 10.1021/acs.est.4c10036

**Published:** 2024-12-09

**Authors:** Layla M. San-Emeterio, Elena Lozano, Victoria Arcenegui, Jorge Mataix-Solera, Nicasio T. Jiménez-Morillo, José A. González-Pérez

**Affiliations:** † Instituto Mediterrâneo para a Agricultura, Ambiente e Desenvolvimento (MED), University of Évora, Núcleo da Mitra, Ap. 94, Évora 7006-554, Portugal; ‡ Grupo de Edafología y Tecnologías del Medio Ambiente GETECMA. Departamento de Agroquímica y Medio Ambiente, Universidad Miguel Hernández, Avenida de la Universidad s/n, 03202 Elche, Alicante Spain; § Instituto de Recursos Naturales y Agrobiología de Sevilla (IRNAS, CSIC)Av. Reina Mercedes, 10, Sevilla 4012, Spain; △ Grupo MOSS, Instituto de Recursos Naturales y Agrobiología de Sevilla (IRNAS, CSIC), Av. Reina Mercedes, 10, Sevilla 4012, Spain

**Keywords:** soil organic matter, glomalin-related soil protein, analytical pyrolysis, wildfire, chemometrics

## Abstract

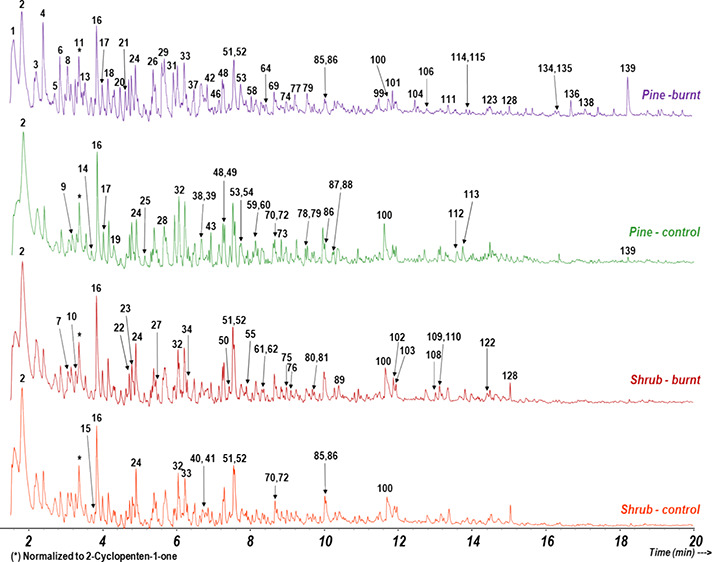

Easily extractable glomalin (EEG) is a fraction of soil organic matter thought to contain mainly glomalin-related soil glycoproteins produced by mycorrhizal fungi. The EEG has an impact on various soil ecological functions, primarily related to soil aggregation formation and stability as well as water repellence. Here, analytical pyrolysis–gas chromatography/mass spectrometry (Py-GC/MS) was used for studying the molecular composition of soil EEG, and a detailed description of the chemical composition is reported. Samples extracted from Mediterranean soils under different vegetation covers (*Pinus halepensis* and shrubland species, *Rosmarinus officinalis*, and *Brachypodium* spp, predominantly), impacted or not by forest fires and collected at different times, were studied. A total of 139 compounds were identified and grouped based on their probable biogenic origin. The EEG chemical composition is dominated by lipids, aromatic compounds, steranes, and hydroaromatics with a remarkable abundance of compounds from plant origin. Significant EEG structural changes can indicate environmental disturbances such as those after a wildfire. The EEG soil organic fraction is found to be a stable and heat-resistant material in nature if soil temperatures remain below 200–250 °C. This study advances the understanding of EEG by providing a detailed molecular characterization and highlighting its role as a stable, heat-resistant component of soil organic matter in Mediterranean ecosystems. The main findings indicate that while EEG is structurally resilient and mostly originates from plant material, its composition is more similar to that of humic acids than to that of glycoproteins.

## Abstract 2

Easily Extractable Glomalin (EEG) was useful for tracing structural changes in SOM given by the vegetation cover and the occurrence of wildfires.

## 1 Introduction

Arbuscular mycorrhizal fungi (AMF) are a type of symbiotic fungi that form mutual associations with the roots of most vascular plants. They are a ubiquitous, dominant group of organisms in soil and make up to 30% of the microbial population.^1^ The AMF play a major role in key soil processes through the production of extracellular compounds and forming extensive hyphae networks.^2,3^

Glomalin, which is also known as glomalin-related soil proteins (GRSP), has been considered a glycoprotein mainly produced by AMF spores and hyphae walls during active colonization of plant roots.^4^ It plays a pivotal role in maintaining soil health, binding soil particles, and contributing to soil structure and stability.^5^ This glycoprotein is assumed to be part of the stable soil organic matter (SOM) pool and significantly contributes to the soil organic carbon pool, accounting for 5–13% of total organic carbon.^6,7^ Glomalin also contributes to the long-term evolution and stability of forest ecosystems by supporting the establishment and succession of plant communities, including trees.^8^ Additionally, GRSP can contribute to reducing the bioavailability of heavy metals in soils by acting as a chelator.^9^ The GRSP can be operationally separated into various fractions based on their extraction from the soil matrix, i.e., easily extractable (EEG) and difficulty extractable (DEG) glomalin.^10^ The EEG is thought to be the newly produced and more active fraction,^11^ while the DEG fraction would correspond to a relatively recalcitrant fraction where glomalin is bound to soil minerals, contrary to EEG that would correspond to a nonbound fraction.^5^ Indeed, the EEG fraction has been proven to increase the stability of soil aggregates, playing hence a relevant role in soil carbon sequestration.^12^

Despite glomalin being such a relevant fraction in SOM and a matter of existing discussion, little is still known about its nature and chemical structure,^13^ to the point that it is still even in question its glycoproteic nature and origin from AMF.^14^ Previous studies have described the glomalin elemental composition: C, 36–59%; H, 4–6%; O, 33–49%; N, 3–5%; P, 0.03–0.1%.^15,16^ Albeit glomalin is widely presumed to be a glycoprotein, thus far supported by recent studies,^12,17^ advanced analytical works including ATR FT-IR spectroscopy, ^13^C CP/MAS, solid-state NMR, X-ray absorption spectroscopy (XANES), Py-FIMS, and proteomics find that GRSP does not resemble a typical glycoprotein. Furthermore, there is no clear evidence that glomalin is an AMF-related protein.^2,14^ Information available on glomalin is contradictory and insufficient. Therefore, further analytical studies are encouraged to enlighten the structure of glomalin and how changes in the soil environment can impact this important SOM fraction.^13^

One of the main disturbing factors affecting soil structure and related properties, hence potentially the structure of GRSP, is the occurrence of wildfires. After a fire, important changes can occur regarding soil physical, chemical, and biological processes^18−20^ that in turn may affect the production of glomalin by affecting AMF.^21^ In this sense, decreases of up to 50% AMF infections have been observed in burned soils.^22−24^ However, the studies of EEG properties immediately after a wildfire under field conditions can be challenging due to multiple factors. As previously reported, measuring glomalin immediately after wildfire is not directly related to fire impact on the AMF community.^23^ Changes in glomalin have been detected with physical soil properties of interest after a wildfire, such as soil aggregation and soil water repellency.^25,26^ However, the response of AMF to fire is highly dependent on the influence of fire on its host plants and cannot be easily predicted.^27^ It has been found that the GRSP content can be used as an indicator of soil heating temperature, which can provide useful information about the severity of fire.^28,29^ Research has shown that the concentration of glomalin in soil is sensitive to the temperature changes caused by heating, even at temperatures as low as 180 °C, and this sensitivity can vary based on the type of soil.^30^

Within this context, in this study, we aimed to achieve the following objectives:

1. To characterize the composition of the EEG fraction at a molecular level using analytical pyrolysis (Py-GC/MS).
2. To study possible variations in composition caused by differing plant covers (pine vs shrubs).
3. To assess the response of EEG to fire and determine if such variations are also related to the seasonality.

## 2 Materials and Methods

### 2.1 Study Site and Soil Sampling

Soils samples were taken from two burned and unburned (control) adjacent forest sites in the locality of Gorga (38°44'56”N; 0°2'32”E), Alicante province (SE Spain). Sampling details and environmental conditions of the area are detailed in ref (23). In summary, the soils are Lithic Xerorthent^31^ developed over Jurassic limestones and under a subhumid Mediterranean climatic condition with a mean annual temperature of 14.6 °C and a 500 mm mean annual precipitation. Further details on other soil physicochemical parameters are listed in Table 1.

**Table 1:** Main Physicochemical Soil Characteristics from the Study Site^a^

						**texture classification: sandy silty loam**		
	**aggregate stability (%)**	**total content of aggregates (%)**	**SOM content (%)**	**pH** (1:25 **w/v, H**_**2**_**O)**	**carbonates (%)**	**sand (%)**	**silt (%)**	**clay (%)**
soil (0–2.5 cm)	84.1 ± 1.3	76.4 ± 0.0	4.2 ± 0.2	7.9 ± 0.1	57.0 ± 5.0	50	41	9

a mean ± standard deviation, *n* = 3.

The vegetation is dominated by *Pinus halepensis* (*P. halepensis*) with an understory of Mediterranean shrub species: *Rosmarinus officinalis* (*R. officinalis*), *Cistus* spp, and *Brachypodium* spp. In July 2011, a part of the area of study was affected by a forest fire that burned ca. 40 ha. Immediately after the fire, plots (1 × 2 m^2^) were set up for monitoring in burned (“fire” or “burnt”) and adjacent control (“control”) unburned areas, underneath *P. halepensis* (hereafter, “pine”) and shrub species (each plot with similar species composition, a mix of *R. officinalis* and *Brachypodium* spp).

The plots were sampled immediately, 4, 8, and 12 months after the fire to extract EEG and to monitor the evolution in the different seasons. Taking into consideration that, mainly due to the low thermal conductivity of the soils, the changes in soil characteristics after a wildfire are restricted to the first topsoil centimeters,^21,32^ surface samples were taken in the first mineral A horizon of soils, down to a depth of 2.5 cm. Each sample was composed of three subsamples randomly taken under the same stem. Samples beneath *P. halepensis* were collected at a 0.5 m distance from the base of trunks, and the subsamples were separated 50 cm from each other. A total of 16 composite soil samples were taken per site: eight in the burned region (four under the influence of pines and four under shrubs) and the other eight samples in the unburned nearby area under comparable conditions, which served as controls. Soil samples were dried at room temperature (20–25 °C) to a constant weight and sieved to fine earth (<2 mm) before analysis. It must be stated that this sieving size has been proven to retain the majority of the glomalin fraction due to its role of binding soil particles and organic matter within this fraction.^33,34^

### 2.2 Glomalin Extraction

Easily extractable glomalin (EEG) was extracted from 0.25 g subsamples with 2 mL of a citric acid buffer, pH 7.0, at 121 °C for 30 min in an autoclave. After the extractions, samples were centrifuged at 3000 rpm for 15 min to remove soil particles. The protein content in the supernatant was determined using a Bradford microassay by reading absorbances at 595 nm in a UV–vis spectrophotometer against a standard curve with varying concentrations of BSA (bovine serum albumin). The method followed is described in detail in refs (5), (23), and (35). Samples were subsequently freeze-dried and preserved at −20 °C until analysis.

### 2.3 Pyrolysis–Gas Chromatography/Mass Spectrometry (Py-GC/MS)

Analytical pyrolysis combined with gas chromatography/mass spectrometry (Py-GC/MS) was performed in solid freeze-dried EEG extracts shortly after extraction. A double-shot pyrolyzer (Frontier Laboratories, model 2020i) coupled to a GC/MS Agilent 6890N system was used. The EEG samples (between 1 and 2 mg) were placed in small deactivated steel crucible capsules and dropped in a preheated (500 °C) microfurnace with an inert He atmosphere for 1 min. The pyrolysis temperature was selected based on the results of an evolved gas analysis (EGA), revealing that the highest release of organic compounds occurs between 400 and 500 °C (Supporting Information, Figure S2). The released pyrolysates were directly injected into the GC/MS for analysis, operating in split mode (split ratio 1:22.3). Both the pyrolyzer and GC inlet lines were heated at 250 °C. The gas chromatograph was equipped with an HP-5 ms-UI, low polar-fused silica capillary column of 30 m × 250 μm × 0.25 μm film thickness. The oven temperature was held at the initial 50 °C for 1 min and was then gradually increased to 100 °C at a rate of 30 °C min^–1^ and from 100 to 300 °C at a rate of 10 °C min^–1^ and finally maintained stable at 300 °C for 10 min. The carrier gas was He at a controlled flow of 1.2 mL min^–1^, and the transfer line from GC to MSD was set at 280 °C. The detector consisted of an Agilent 5973 MSD, and mass spectra were acquired at a 70 eV ionizing energy. Compound assignment was achieved by monitoring diagnostic ions for the main homologous series, via low-resolution MS and by comparison with published and stored (NIST and Wiley libraries) data. A final semi, relatively quantitative assessment of the identified compounds was done based on the integration of each pyrolysis product peak, excluding minor compounds (<0.2% total chromatographic area) and the proportions calculated as percentages of the total chromatographic area as described in ref (36).

### 2.4 Statistical Analysis

Normality and homoscedasticity were checked prior to any statistical analysis using the Kolmogorov–Smirnov test and the Levene test, respectively. Data did not meet the normality or homogeneity requirements even after a log transformation was applied, and an analysis of variance (ANOVA) was found to be inappropriate for the available data. Therefore, nonparametric tests were used for treatment comparisons, i.e., the Kruskal–Wallis test along with a Dunn post hoc test for multiple nonparametric comparisons and the Mann–Whitney *U* test. These statistical analyses were made with a 95% confidence level using the statistical package SPSS 20.0 (SPSS, Inc., Chicago, USA).

Lastly, a factor analysis of mixed data (FAMD) was applied as a function of principal component analysis (PCA), where both quantitative and qualitative data were analyzed.^37^ In this case, FAMD was used to assess the effect of categorical variables (“fire” and “vegetation”) on the average relative abundance of each family compound identified by pyrolysis. RStudio (version 2022.02.3) and “FactoMineR” and “ggplot2” packages were used for the graphical representation of multivariate data.

The potential influence of the different factors on the glomalin composition was explored with canonical discriminant analysis (CDA) using SPSS 20.0. In this case, the relative abundances of the different families of pyrolytic compounds were used as descriptors (independent variables). On the other hand, the dependent variables consisted of the different environmental factors that may define or condition the glomalin composition (vegetation cover, fire, and season of the year). The different qualitative sample descriptors for each factor were, for the vegetation cover, “pine” and “shrub”; for the occurrence of fire, “burnt” and “control”; and lastly, for the season where sampling was conducted, “spring”, “summer”, and “winter”.

### 2.5 Van Krevelen Diagrams

Attending to the large dimension of the pyrolysis data matrix, to detect minor differences in EEG composition and the effects of the different treatments, the analysis of EEG pyrolysis was complemented with a graphical-statistical method. This consisted of a revised version of the classical van Krevelen^38^ adapted for the representation of large chromatographic data sets as described in ref (39). The plots represent the H/C and O/C ratios calculated from the empirical formulas of the identified molecules (*x* and *y* axes), while the relative abundances are plotted on the *z*-axis. Deviations in pyrolytic compound compositions between samples can also be highlighted by generating subtraction surfaces with peaks and valleys revealing concentration and selective depletion, respectively, of EEG structural domains under the differing conditions compared (vegetation and fire).

An EEG model sample was calculated with the average molecular composition of all of the samples. This model sample was then used to compare the differences with the main families of each EEG sample using subtraction density van Krevelen diagrams. With this approach, we were able to visually compare the chemical composition of the various EEG samples extracted from the different situations (vegetation and fire). Furthermore, the relative abundances of the organic compounds released after pyrolysis in the different treatments were compared using Student’s *t* test to assess the significance of variations in the percentages of each component between the average samples and the model sample. Microsoft Excel 2019’s function = T.TEST (array1, array2, tail: 2, type: 3) was used to compute Student's *t* test. The chemical compounds significantly different (*p* < 0.05) are represented as a subtracted density van Krevelen plot in the form of an overlaid contour diagram on the density map. The colored levels indicate the different relative abundances of the organic families.^40,41^

## 3 Results and Discussion

### 3.1 Analytical Pyrolysis of EEG (Py-GC/MS)

**Figure 1:**
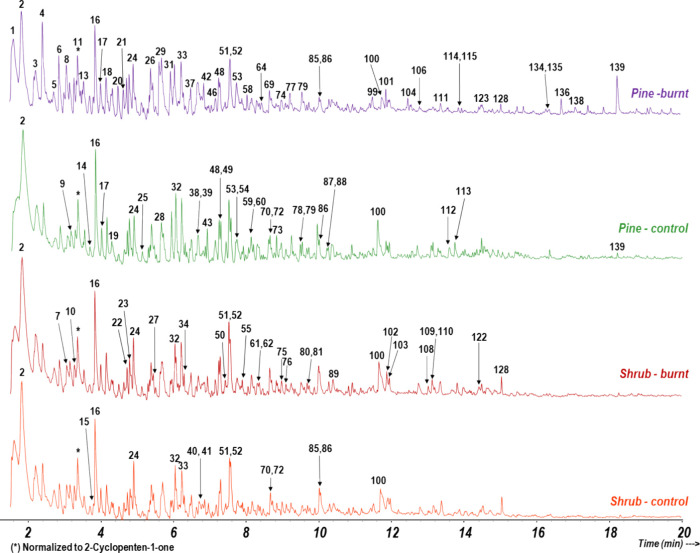
Selected pyrochromatograms of EEGs representative for each vegetation cover, control, and burnt. Labels on the peaks are referred to Table S1, and the pyrochromatograms are normalized to the high of peak 11 (cyclopentenone).

A total of 139 different compounds were released and identified in the EEG fractions after analytical pyrolysis (Supporting Information, Table S1). As observed in the example pyrochromatograms shown in Figure 1, there was no clear difference, and a high degree of similarity was found among EEG samples obtained from various plant covers, regardless of whether they were affected by forest fire or not. This points out EEG as a uniform SOM fraction given its molecular composition.

**Figure 2:**
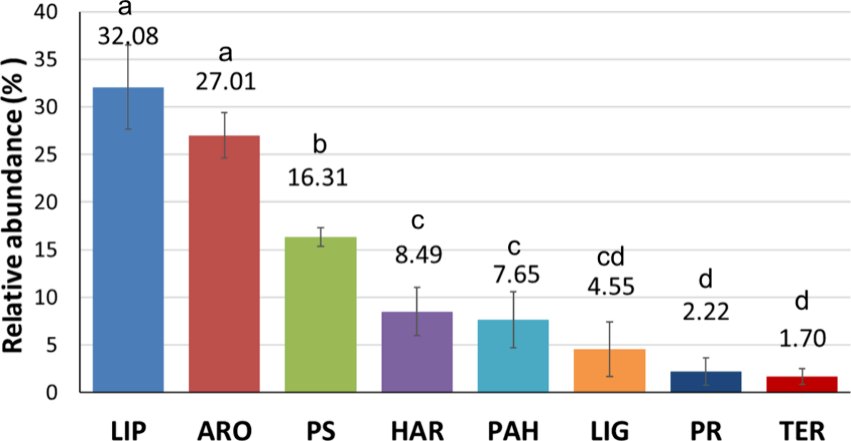
Composition of the different biogenic chemical groups identified in the EEG extracts. This is an average sample of the compounds identified in all the EEG extracts. Lipids (LIP), aromatics (ARO), polysaccharides (PS), hydroaromatics (HAR), polycyclic aromatic hydrocarbons (PAH), lignin methoxyphenols (LIG), proteins and peptides (PR), and terpenes (TER). Error bars correspond to the STD (*n* = 16), and families of compounds sharing the same letter are not significantly different.

The compounds identified in the pyrochromatograms could be categorized into eight main families: unspecific aromatic compounds (ARO), hydroaromatic compounds, mainly steranes (HAR), lignin-derived (methoxyphenols) (LIG), lipids (LIP), polycyclic aromatic hydrocarbons (PAH), protein-derived (PR), polysaccharide-derived (PS), and terpenes (TER), the average proportions of which in the EEG pyrolysates are represented in Figure 2. Only the LIP, ARO, PS, HAR, and PAH components accounted for more than 92% of the relative abundance of all compounds identified, while the remaining 8% was composed of variable abundances of LIG, PR, and TER.

The results obtained by Py-GC/MS were also confirmed by infrared spectroscopy (FT-IR) (Supporting Information, Figure S3). In line with the previous results, the FT-IR analysis showed no appreciable differences between the burned and unburned samples or among the various types of vegetation covers. Consequently, it was necessary to analyze in more detail the pyrolysis results, aided by chemometric and graphical-statistical analyses.

#### 3.1.1 Alkyl and Lipid Compounds (LIP)

This group of pyrolysis compounds was the most abundant group detected (32.08 ± 4.44%), mainly short, cycled hydrocarbons (cyclopentadienes, cyclohexadienes, and cyclohexenes) and midlong alkyl straight chains. The *n*-alkane distribution followed a well-resolved bimodal series (C_10_ to C_34_) with maxima in C_13_ or C_15_ and C_29_ or C_31_ (Supporting Information, Figure S4). This was possible by monitoring the ion *m*/*z* 57, which has been documented to be a reliable diagnostic ion for *n*-alkanes at a 70 eV ionizing energy.^42^ No clear carbon number predominance was found in the short chain range (<C_24_) but a conspicuous odd-chain carbon number predominance as observed in the longer moieties (>C_25_). This indicates two distinct probable origins for the alkanes present in the EEG: microbial for the short-mid chains^43^ and a plant origin from epicuticular waxes for the long ones.^44,45^ Some other aliphatic compounds such as saturated and unsaturated fatty acids and fatty acid methyl esters were found as part of SOM functional groups: *n*-hexadecanoic and *n*-octadecenoic acids and methyl esters.

#### 3.1.2 Aromatics (ARO)

This was the next family of compounds in abundance (27.01 ± 2.35%), mainly low-molecular-weight alkylbenzenes (C_1_–C_3_), phenol and alkylphenols (C_2_–C_3_), and indene and methylindene. However, the origin of these compounds is unknown, making them poor biomarkers. Nevertheless, short alkyl side-chain phenols may have come from plant biomass through the removal of methoxyl units from lignin monomeric units^46^ or from plant resin terpenes^47^ or tannins.^48^

#### 3.1.3 Carbohydrate-Derived (PS)

The third group of relevance was PS (16.11 ± 0.97%), with low-molecular-weight cyclic ketones (cyclopentanones and cyclopentenones) and alkyl derivatives and furans as the more abundant compounds. These are considered typical thermal degradation products from carbohydrates and usually in soil organic fractions from plant holocelluloses (cellulose and hemicellulose) but also from chitin.^49^ In addition, the extracellular polymeric substances (EPS), which microbes release to build biofilms, may be the source of furan compounds, such as furan, 2-ethyl and benzofuran, 2-methyl.^50^

#### 3.1.4 Hydroaromatics (HAR)

This group of compounds (8.49 ± 2.54%) is mainly composed by alkyl-substituted indenes and azulenes and hydronaphthalenes, anthracenes, and phenanthrenes, with a probable origin from natural resins, many of which have a hydroaromatic structure.^51^

#### 3.1.5 Polycyclic Aromatic Hydrocarbons (PAH)

This group (7.65 ± 2.97%) included mainly naphthalene, fluorene, phenanthrene, anthracene, and alkyl derivatives. These aromatic hydrocarbons have a probable origin in the incomplete burning of biomass by, i.e., forest fires.^52^ However, no remarkable difference was observed after the fire, which is contrary to what was expected. The natural PAH retene and a few derivatives, a known conifer biomarker,^53^ were mainly found under the pine vegetation.

#### 3.1.6 Lignin-Derived (LIG)

Phenolic structures including *p*-hydroxyphenyl (H) and guaiacyl (G) units were also found (4.55 ± 2.88%) in the EEG pyrolysate. These are well-known plant biomarkers, subunits from polyphenolic biopolymers like lignins.^54^

#### 3.1.7 N-Containing Compounds (PR)

Nitrogen-bearing compounds that may be related to N-rich biopolymers were not abundant, accounting for only a minor proportion in the EEG pyrolysates (2.22 ± 1.45%). These mainly included indole and a number of pyrrole derivatives. However, other biomarkers that could be undoubtfully related to proteins, such as cyclic dipeptides (2,5-diketopyperazines),^55^ were not found. This observation is further supported by both complementary analysis, EGA and FT-IR, which did not show significant protein-related absorption bands, indicating the absence or low presence of intact proteins in the samples (Supporting Information, Figures S2 and S3, respectively). This is such a remarkable finding since, contrarily, EEG has been considered to be mainly composed by N-linked glycoprotein from arbuscular mycorrhizal fungi.

#### 3.1.8 Terpenes (TER)

This was the less abundant class of compounds detected in EEG pyrolysates (1.70 ± 0.83%). These compounds were unsaturated hydrocarbons produced predominantly by plants and, particularly, by conifers. The most probable origin is plant resins and plat volatile components (essential oils).

In general, the EEG fraction was found to be highly aromatic, with a low protein content and a marked plant origin as indicated by lignin and alkyl markers. However, a limited contribution from microbial carbohydrates and fungal (chitin) contributions are not ruled out. In general, this study of the EEG chemical structure using analytical pyrolysis is consistent with previous findings using other spectroscopic techniques like FT-IR and ^13^C PMAS NMR,^16,56^ which showed that glomalin extracts are highly aromatic (C-aryl) and rich in acid (C-carboxy) groups that do not resemble a typical glycoprotein but are close to that of a humic acid.

### 3.2 Effect of Plant Cover, Fire, and Time of Sampling on EEG Composition

**Figure 3:**
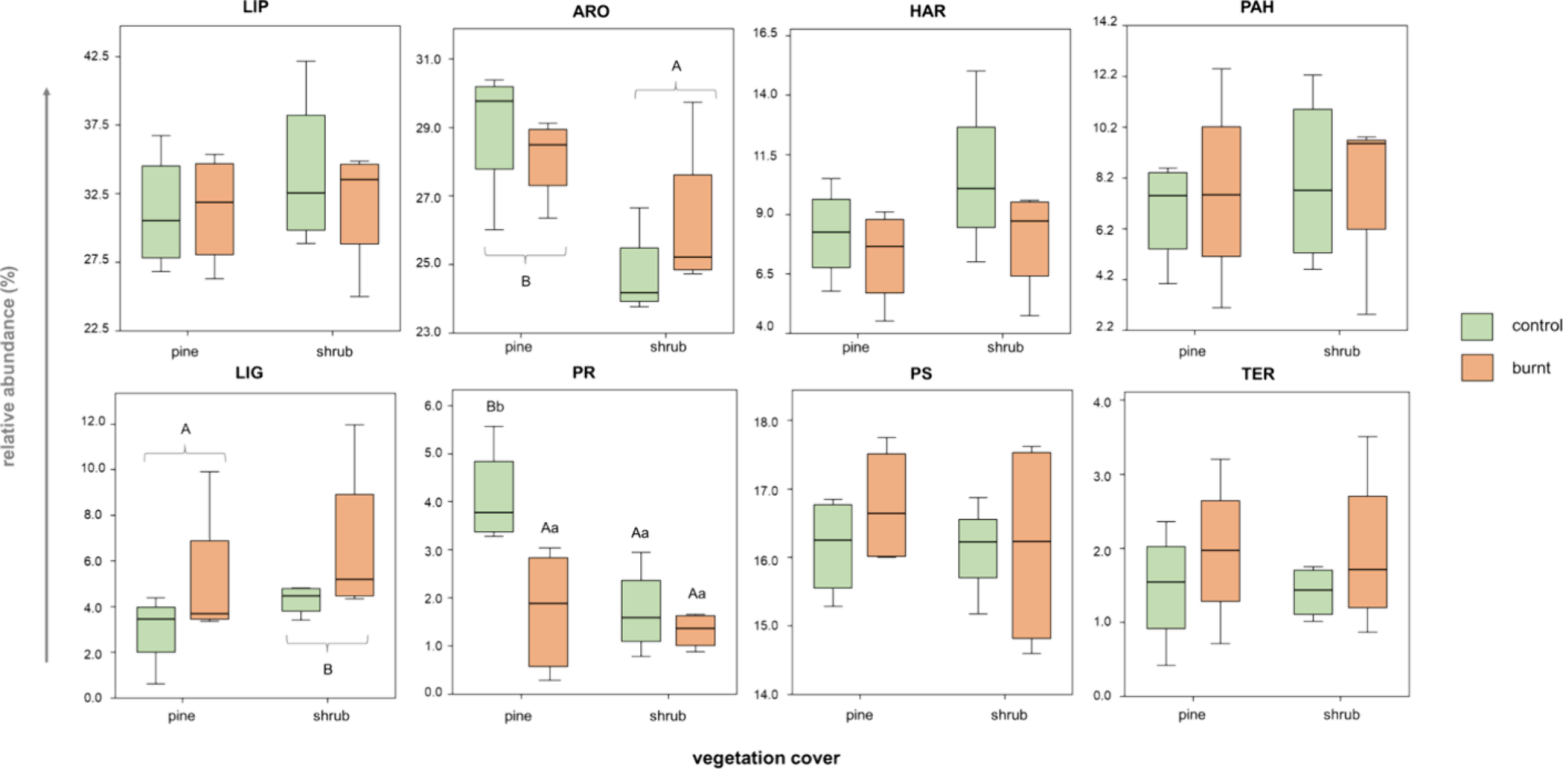
Distribution of the relative abundance (%, *n* = 4) of each biogenic group identified by Py-GC/MS. Uppercase letters represent significant differences between vegetation covers, whereas lowercase letters represent significant differences between control and burnt plots (*p* < 0.05).

The distribution of the relative abundance of each biogenic group did not show any direct effect of the sampling season; hence, the data distribution was mainly studied according to the type of vegetation cover and the effect of the fire (Figure 3). Biogenic groups in EEG affected by the vegetation coverage were ARO and LIG with the former significantly more abundant under pine (*p* = 0.01) and the latter under shrub (*p* = 0.05). These differences observed under the two vegetation covers could be due to the fact that litter inputs may affect differently glomalin under shrub cover due to the presence of a thinner, less woody vegetation.^57^ Another reason could be that aromatic structures from resins and lignins tend to accumulate more under pine trees,^58^ especially after a wildfire,^59^ which in turn is reflected in the EEG chemical structure.

A significant interaction was also detected between vegetation cover and fire, with a reduction of PR compounds under burnt pine vegetation (*p* = 0.03, Figure 3). This could be explained by differences in fire temperatures as influenced by the spatial distribution and type of vegetation.^60^ The range of maximum temperatures in shrublands (300–700 °C) tends to be higher than in mature tree forests (200–300 °C),^61^ which could explain the differences found between burned plots for PR and LIG compounds (Figure 3). In fact, a relatively higher abundance of LIG compounds was also observed under fire conditions, though not statistically significant at *p* = 0.05. It has been also reported that fire preferentially removes thermolabile biogenic materials with a selective preservation of lignin, which may be adding to this observed enrichment.^62^

**Figure 4:**
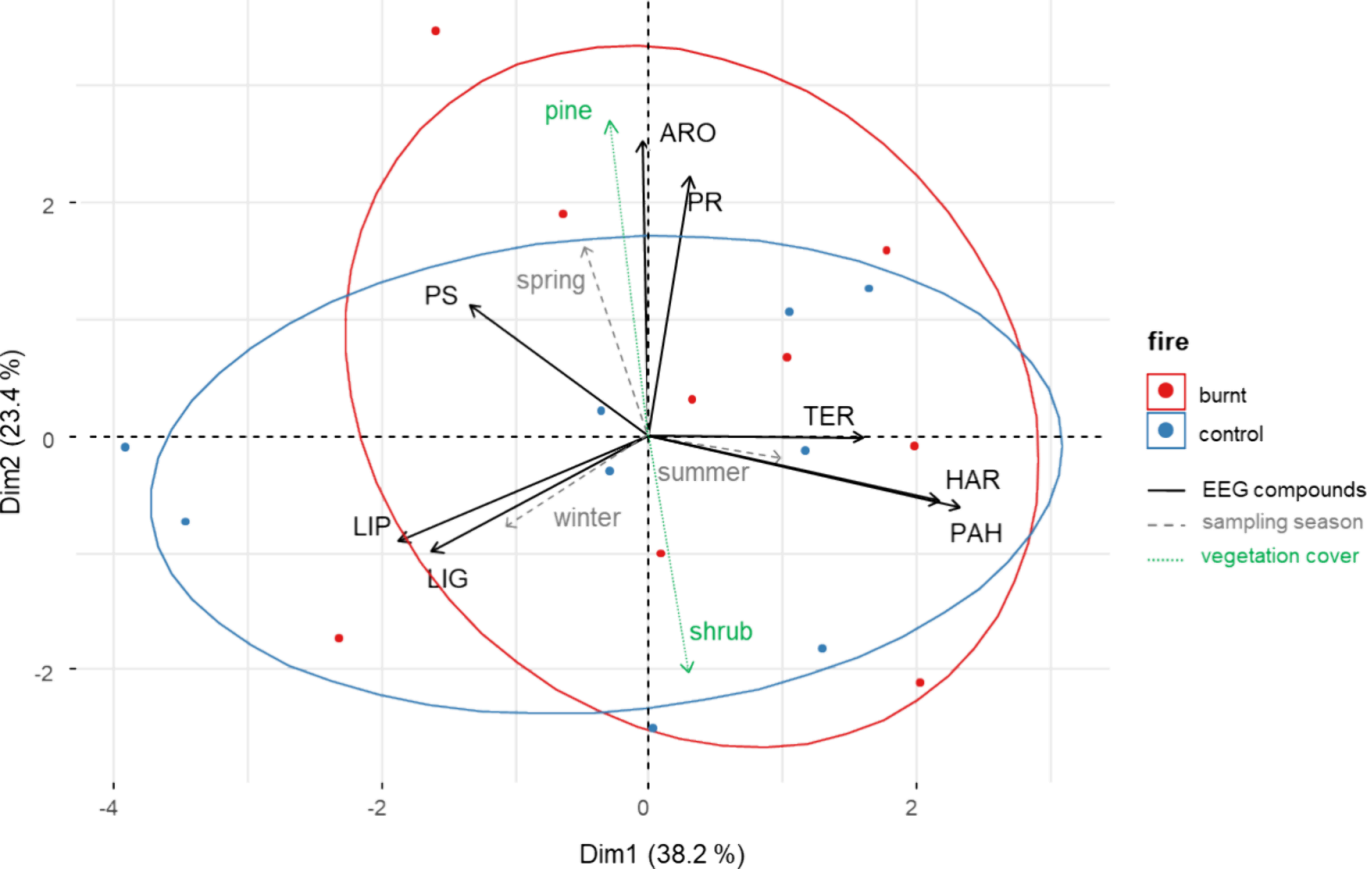
Factor analysis of mixed data analysis using mean values of families of EEG pyrolytic compounds as quantitative factors (solid black arrows), supplementary factors as sampling seasons (dashed gray arrows), and vegetation covers (dotted green arrows), as well as fire conditions (solid ellipses) as categorical factors. Ellipses indicate a 95% confidence level (CI).

The application of factor analysis of mixed data (FAMD) allowed assessing the effect of vegetation cover, fire, and time of the year (season) as qualitative variables, on the main groups of biogenic compounds. Up to 61.6% of the total variance is explained in a 2D plot (Figure 4). The first component (Dim 1) is defined by high loadings of PAH and HAR and low loadings of PS and LIP (Table 3). Also, the effect of the season is greatly impacted on this dimension, especially with negative scores related to the winter season. The highest discriminant values in the second component (Dim 2) were due to ARO compounds and the type of vegetation. Although clusters of fire and control effects are revealed, the size of these clusters is not intuitively differentiated among the diverse EEG compounds, confirming the scarce significant differences previously evaluated.

**Table 2:** Total Variance Explained and Other Mathematical Factors Associated with the Discriminant Function Analysis of EEG Composition and Selected Environmental Factors

	**function**	**eigenvalue**	% variance	cumulative %	**canonical correlation**	**Wilks’ lambda**	**chi-square**	**df**	**sig.**
(A) season	1	2.497	62.5	62.5	0.845	0.117	20.362	16	0.204
	2	1.439	37.5	100	0.768	0.410	8.468	7	0.293
(B) fire and vegetation cover	1	3.867	60	60	0.891	0.054	26.211	24	0.343
	2	2.092	33.8	93.8	0.823	0.265	11.968	14	0.609
	3	0.223	3.6	100	100	0.818	1.810	6	0.936

**Table 3:** Factor Analysis of the Mixed Data Dimension Matrix^a^ for the Main EEG Pyrolysis Products in the Different Sampling Seasons, Vegetation Cover, and Fire Occurrence

**variables**	**Dim 1**	**Dim 2**
PS	–0.52	
PR		
ARO		0.82
TER		
HAR	0.75	
PAH	0.81	
LIP	–0.67	
LIG		
season	0.71	–0.58
vegetation cover	0.87	–0.63
fire	–0.57	
eigenvalue	3.1	1.9
proportion variance (%)	38.2	23.4
cumulative variance (%)	38.2	61.6

a Coordinates of the variables with the FAMD factors. Only those factors with significant contributions (*p* < 0.05) are shown.

**Figure 5:**
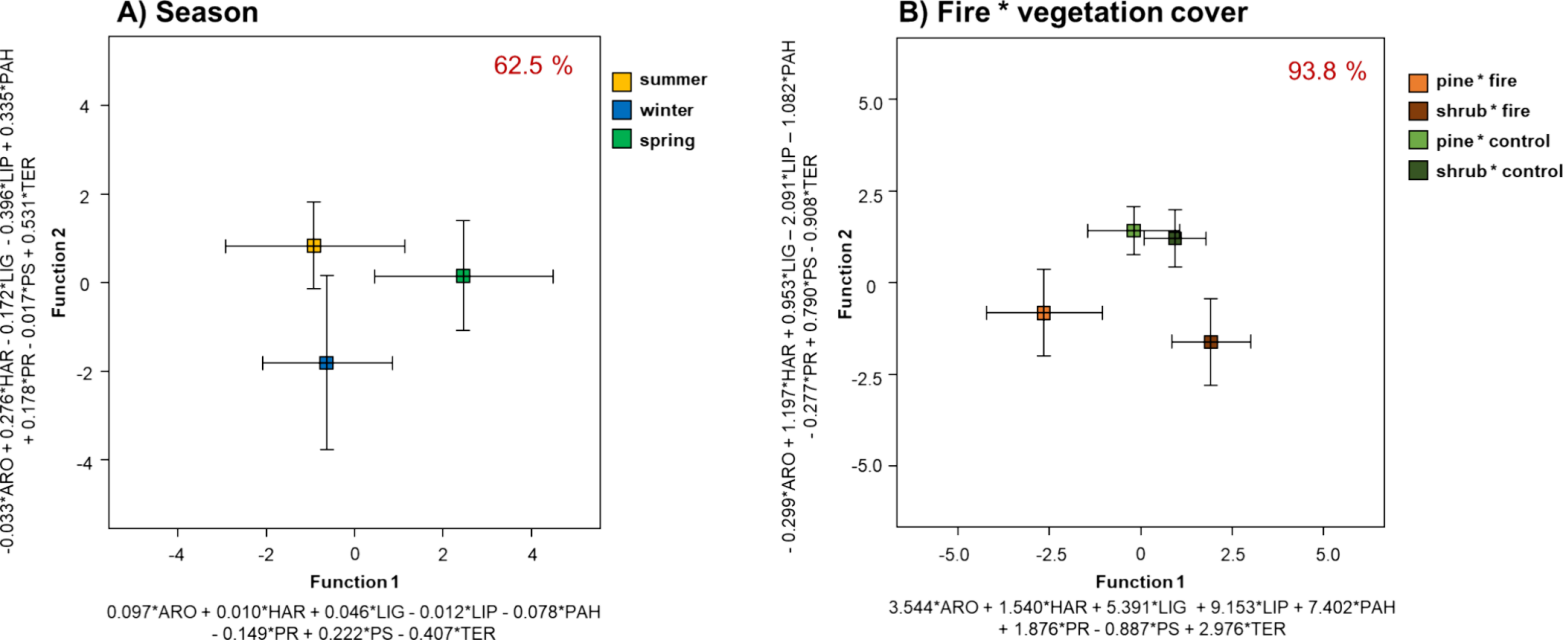
Biplot of discriminant function analysis for separation of EEG composition according to the different environmental factors: (a) sampling season and (b) combined effect of fire and vegetation cover. The coefficients of the discriminant functions are shown on the axes. The percentage of EEG compounds correctly classified in the different groups is shown at the top right corner (%).

A graphical discriminant analysis (CDA) was also performed to deepen the feasible effects of these environmental factors (Figure 5). Attending to the season of the year, it was possible to explain a total of 62.5% of the data variance and 93.8% of the variance for the interactive effect of the vegetation cover and the effect of fire (Table 2). Regarding the effect of the sampling season, a high overlap with no significant differences was observed (Figure 5A). The combined effect of fire and vegetation cover (Figure 5B), although not statistically significant (*p* < 0.05), could distinguish differences due to fire depending on the pine or shrub cover (*p* = 0.091). Whereas no effects on the molecular structure were observed between the EEG extracted from control soils under pine or shrubs, fire seems to have played an effect on EEG composition. Again and in line with the FAMD results, the high loadings of the corresponding CDA functions (function 1) point to the fact that the ARO, LIP, and PAH compound groups are indicative of fire disturbance on the EEG chemical structure.

### 3.3 Van Krevelen Diagrams

Surface density plots constructed over van Krevelen diagrams were especially useful for monitoring and summarizing the different chemical transformations exerted by fire. The 3D van Krevelen, along with atomic H/C and O/C values, also displays a third dimension based on the proximity to the basal plane, representing clusters of compounds with similar stoichiometry. Therefore, the different families of organic compounds and their relative abundances are easily distinguishable in distinct areas of the diagram^63^ (Supporting Information, Figure S5).

**Figure 6:**
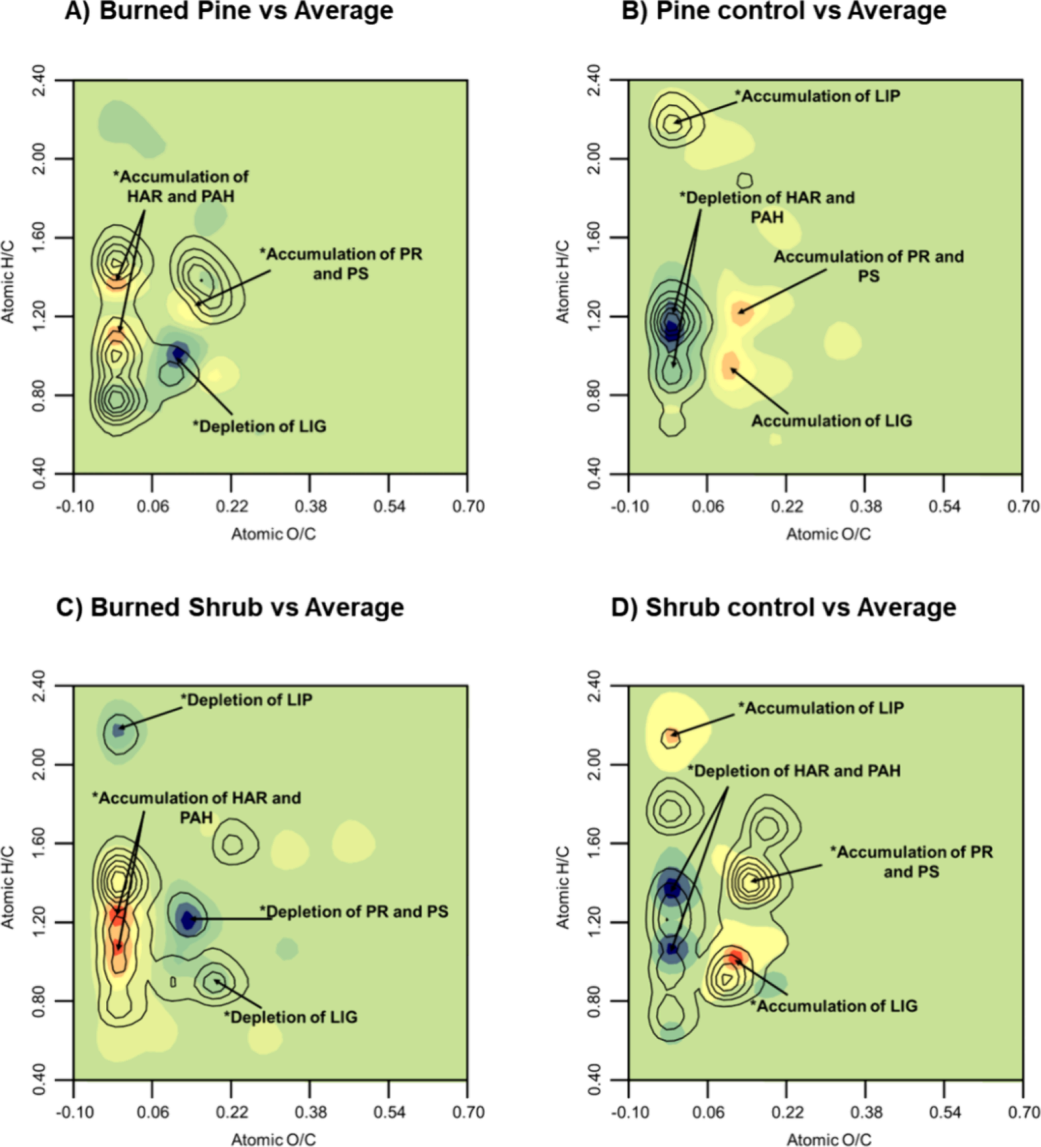
(A–D) Subtraction density van Krevelen plots illustrate the difference between the abundances of the main organic families in the average molecular composition of all samples (model sample) and that corresponding to each of the individual samples, represented in the space defined by the H/C and O/C atomic ratios (positive values are shown in red colors, and negative values are shown in blue colors). The significantly different compounds (“*”, *p* < 0.05) of each sample were plotted on the contour diagrams superimposed to the subtraction density van Krevelen diagrams.

The subtraction of van Krevelen plots obtained by subtracting the values of the different compounds in samples under different vegetation covers (pine and shrub) and scenarios (fire-affected and control) showed statistically significant differences (*p* < 0.05). This is detected when overlapping the contour map depicting Student *t* values with the compound concentration subtraction ones between sets of samples with different vegetations and impacts of fire. Burned pine was characterized by a significant accumulation of aromatic and condensed compounds and a slight accumulation of oxygenated compounds such as protein-derived and polysaccharides (Figure 6A). In addition, the burned pine EEG shows a significant depletion of lignin-derived methoxyphenol compounds (LIG). In contrast, the pine control sample was dominated by a significant accumulation of lipid-like compounds and a significant depletion of aromatic compounds (Figure 6B). There was also an accumulation of proteins, polysaccharides, and methoxyphenol compounds but not significant (*p* > 0.05). In the case of EEG from shrub, the fire-affected samples also showed a significant accumulation of hydroaromatic and condensed (PAHs) compounds but significant depletion not only of methoxyphenols but also of lipids, protein-like, and polysaccharide-derived compounds (Figure 6C). The remarkable depletion of fresh material (LIG methoxyphenols) observed equally under both vegetation covers, together with an increase of naphthalenes and alkyl benzenes, may reflect an external input of partly charred material with altered lignin (defunctionalized methoxyphenols), as described for burnt topsoils.^64,65^ The observed accumulation of newly formed polycyclic compounds has been widely established to be associated with an input of charred materials produced during the burning of biomass.^52,66^

Finally, control shrub samples showed a significant accumulation of methoxyphenols, proteins, polysaccharides, and lipid-like compounds (Figure 6D). In addition, these samples presented a significant depletion of aromatic and condensed compounds, as observed for their homologues under pine. This significant increase in N-containing compounds observed could be attributable to differences in soil texture and organic matter and hence in N accumulation, which could turn into a significant N retention as previously found in semiarid shrublands.^67^

The different trends observed point to the fact that the molecular composition of EEG is directly and significantly conditioned by the factors studied here, vegetation cover and fire. The graphical-statistical tool used here has been shown to be useful in identifying relevant biomarkers surrogated to the effect of vegetation and environmental impacts, i.e., fire in the chemical structure of soil organic fractions.^37^

## 4 Conclusions

This study is the first to use Py-GC/MS to investigate the structural changes associated with EEG in different vegetation covers and identify significant molecular changes that occur after a wildfire. According to our findings, the chemical structure of the EEG does not resemble that of a glycoprotein, as previously thought. Several biomarkers, which could be traced back to plants, are present in the EEG structure. However, we could not rule out a limited contribution from microorganisms.

Various factors, such as soil temperature, structure, vegetation, and time of the year, can impact the amount of EEG present in soil. However, after examination of the distribution of the various organic chemical families, it has been observed that the molecular structure of EEG remains relatively constant. This suggests that EEG is a consistent component of SOM, similar to humic acids. Nonetheless, it is found that the vegetation cover significantly influenced the EEG molecular structure as did the passage of fire. Albeit the vegetation cover and occurrence of wildfire may affect the EEG composition, the similarity found among the control plots indicates that the EEG is structurally homogeneous and probably resilient at temperatures not exceeding 200–250 °C.

Future studies should address soil mineralogy and AM fungal mycelium density to build on these findings and provide a more comprehensive understanding of the factors influencing EEG composition and its role within the SOM.

## Supporting Information Available

(Table S1) Pyrolysis products of EEG from soils affected and unaffected by forest fire, under pine and shrub coverage and different times of the year; (Figure S1) some representative images of the study area and sampling plot examples; (Figure S2) evolved gas analysis (EGA) curves from the bulk soil sample and glomalin extract; (Figure S3) Fourier transform infrared (FT-IR) spectra of glomalin extracts from unburned and burned soil samples collected under pine and shrub vegetation; (Figure S4) single-ion monitoring chromatograms for the alkane series (*m*/*z* 57) released after EEG pyrolysis extracted from soils under pine and shrubs, affected and not affected by fire and at different times of the year; (Figure S5) 3D van Krevelen diagrams that display the pyrolysis products of EEG, characterized by their H/C (*x*), O/C (*y*) atomic ratios, and relative abundances (*z*), for control pine, burned pine, control shrub, and burned shrub; additional comments on Figure S3 (PDF)

## Abbreviations

AMF: arbuscular mycorrhizal fungi
GRSP: glomalin-related soil proteins
SOM: soil organic matter
EEG: easily extractable glomalin
Py-GC/MS: pyrolysis coupled to gas chromatography–mass spectrometry
FAMD: factor analysis of mixed data
CDA: canonical discriminant analysis
PS: polysaccharides
PR: proteins and polypeptides
LIP: lipids
PAH: polycyclic aromatic hydrocarbons
HAR: hydroaromatics
ARO: nonspecific aromatic compounds
LIG: lignin and polyphenols
TER: terpenes
