# Supplementary material for: Soil-Easily Extractable Glomalin: An Innovative Approach to Deciphering Its Molecular Composition under the Influence of Seasonality, Vegetation Cover, and Wildfire
Source: Environ Sci Technol. 2024 Dec 9;58(51):22624–34. doi: 10.1021/acs.est.4c10036 (PMC11673091; doi:10.1021/acs.est.4c10036)
Supplement: Supplementary file 1 — es4c10036_si_005.pdf [file es4c10036_si_005.pdf]

Supporting information

Soil Easily Extractable Glomalin: An innovative approach for deciphering its molecular composition under the influence of seasonality, vegetation cover, and wildfire.

Layla M. San Emeterio†, Elena Lozano‡, Victoria Arcenegui‡, Jorge Mataix-Solera‡, Nicasio T. Jiménez-Morillo§, José A. González-Pérez§**

†University of Évora, Instituto Mediterrâneo para a Agricultura, Ambiente e Desenvolvimento (MED), Núcleo da Mitra, Ap. 94, 7006-554 Évora, Portugal.

§Instituto de Recursos Naturales y Agrobiología de Sevilla (IRNAS, CSIC) Av. Reina Mercedes, 10, 4012 Sevilla, Spain.

‡Grupo de Edafología y Tecnologías del Medio Ambiente GETECMA. Departamento de Agroquímica y Medio Ambiente, Universidad Miguel Hernández, Avenida de la Universidad s/n, 03202 Elche, Alicante, Spain.

Summary: 15 pages, 5 figures, 1 table.

Figure S1. Some representative images of the study area and sampling plot examples. From top to bottom and left to right. 1. Landscape of control area. 2. Landscape of immediately burned area. 3. Control sampling site underneath pine. 4. Burned sampling site underneath pine. 5. Control sampling site underneath shrub. 6. Burned sampling site underneath pine. All pictures were taken just after the wildfire occurred by J. Mataix-Solera – page S3

Figure S2. Evolved Gas Analysis (EGA) curves from a) bulk soil sample and b) glomalin extract – page S4.

Figure S3. Fourier-Transform Infrared (FT/IR) spectra of glomalin extracts from unburned and burned soil samples collected under pine and shrub vegetation – page S5.

Figure S4. Single ion monitoring chromatograms for the alkane series (*m/z* 57) released after EEG pyrolysis extracted from soils under pine and shrubs, affected and not affected by fire and at different time of the year. Cn indicates the number of carbons in the alkyl chain – page S8.

Figure S5. 3D van Krevelen diagrams that display the pyrolysis products of EEG, characterized by their H/C (x), O/C (y) atomic ratios and relative abundances (z), for a) control pine; b) burned pine; c) control shrub and d) burned shrub – page S9.

Table S1. Pyrolysis products[1] of EEG from soils affected and unaffected by forest fire, under pine and shrub coverage and different time of the year – page S10.

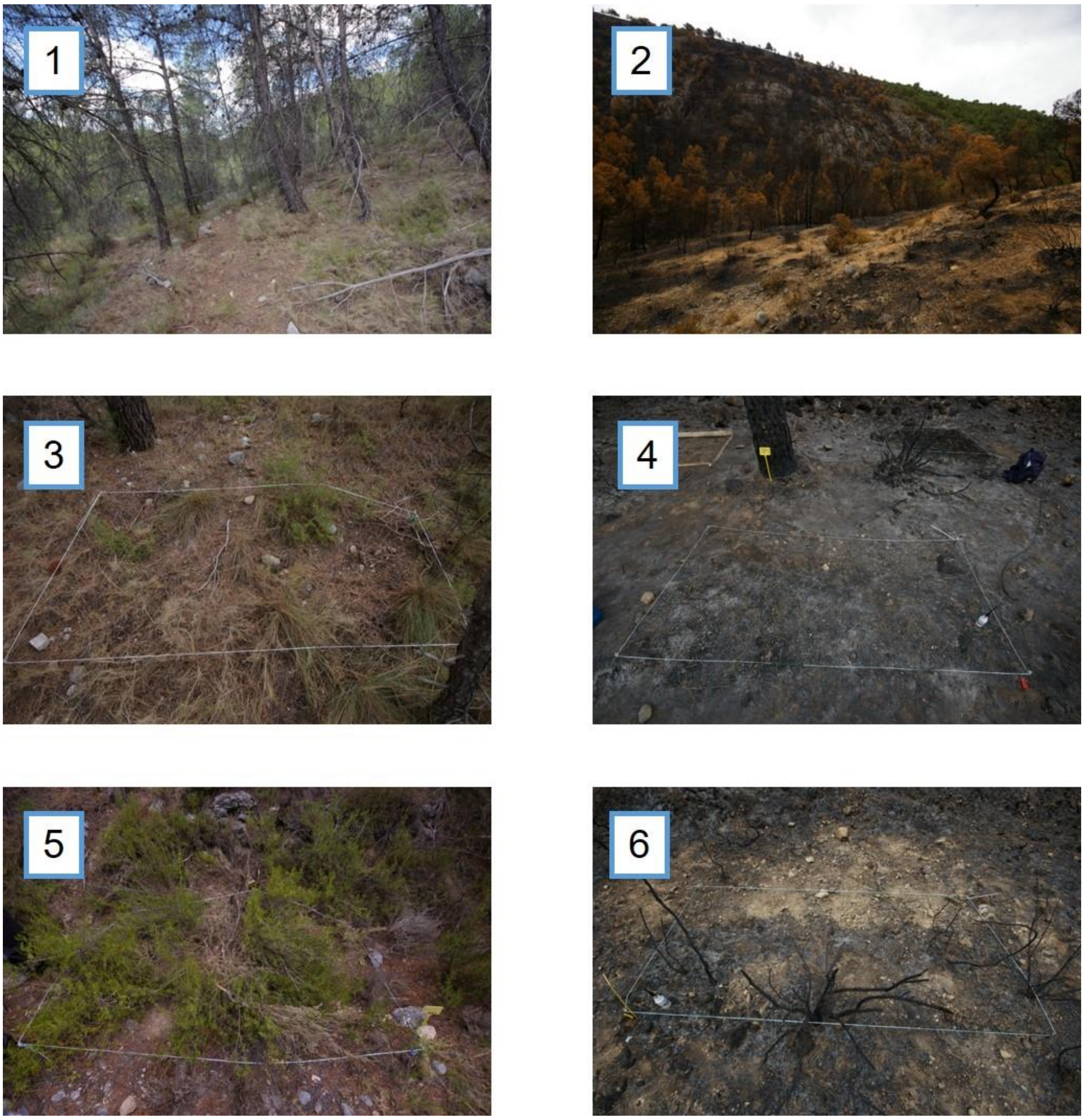

Figure S6. Some representative images of the study area and sampling plot examples. From top to bottom and left to right. 1. Landscape of control area. 2. Landscape of immediately burned area. 3. Control sampling site underneath pine. 4. Burned sampling site underneath pine. 5. Control sampling site underneath shrub. 6. Burned sampling site underneath pine. All pictures were taken just after the wildfire occurred by J. Mataix-Solera.

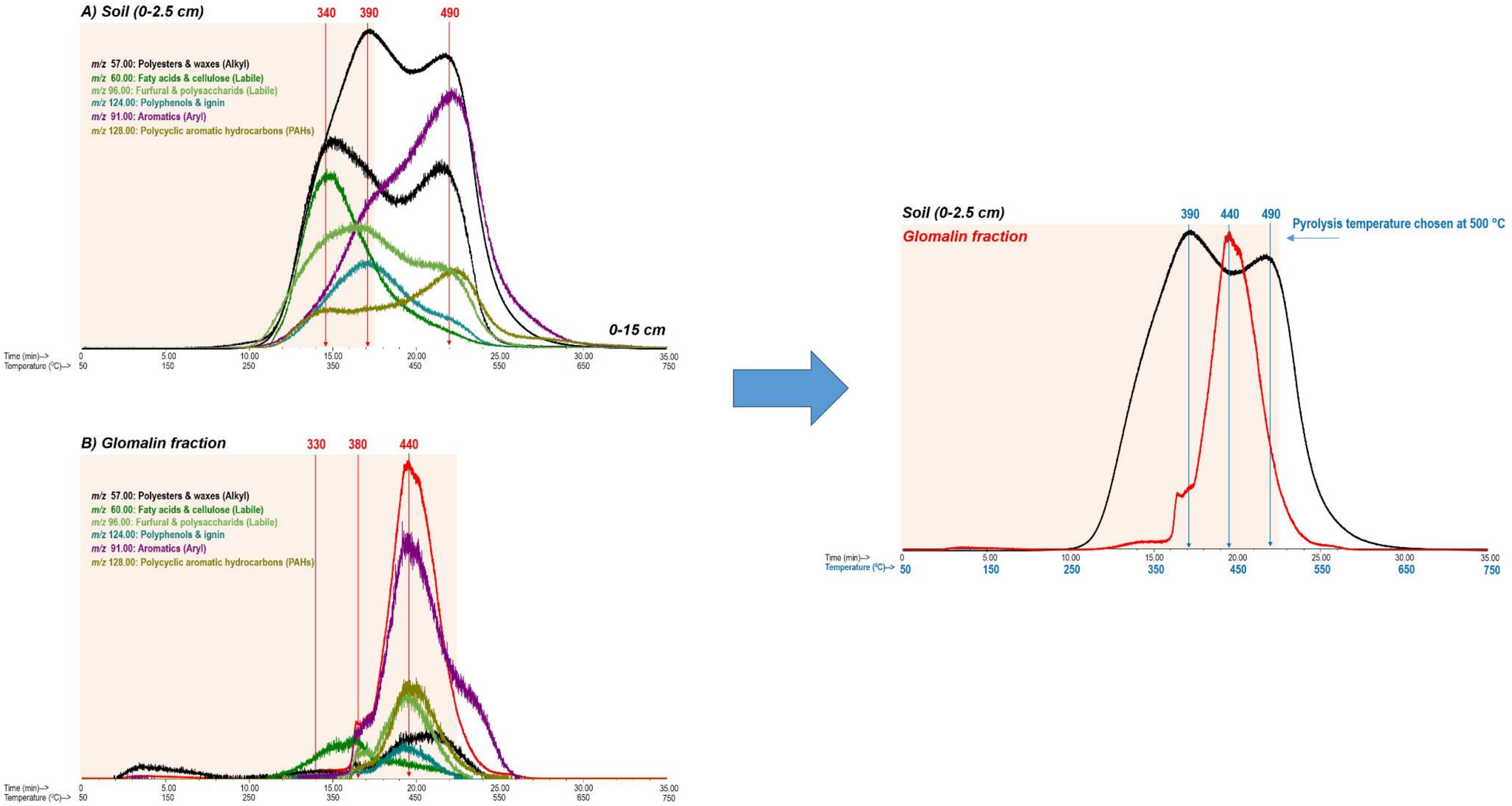

Figure S7. Evolved Gas Analysis (EGA) curves from a) bulk soil sample and b) glomalin extract.

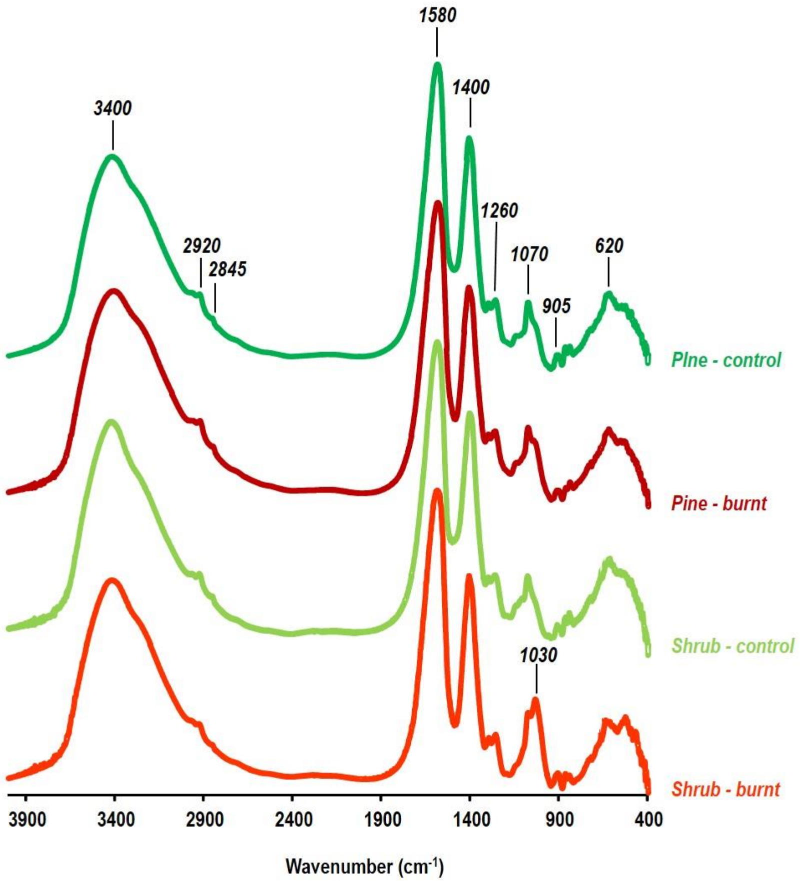

Figure S8. Fourier-Transform Infrared (FT/IR) spectra of glomalin extracts from unburned and burned soil samples collected under pine and shrub vegetation.

Additional comments: Fig. S3 illustrates the average Fourier-transform infrared (FT/IR) spectra of glomalin extracts from unburned and burned soil samples collected under pine and shrub vegetation. The generated infrared spectra show a remarkable similarity between burned and unburned samples, as well as between those collected under pine and shrub vegetation. Therefore, the use of the infrared spectroscopy did not allow the identification of significant differences between burnt and control samples, as well as between different vegetation covers. This investigation demonstrates the necessity of using more sophisticated analytical methods, such as analytical pyrolysis (Py-GC/MS). As depicted in Fig. S1, all spectra display the same bands with identical intensities, except for the EEG under shrub coverage in wildfire, which exhibited a band at 1030 cm^{-1}, corresponding to C–O stretching (polysaccharides[1]). Among the primary bands, a broad band at 3400 cm^{-1} can be identified, which may correspond to the stretching vibrations of bonded and non-bonded hydroxyl groups[2]. The bands at 2920 and 2845 cm^{-1} represent the aliphatic C–H vibrations of aliphatic methyl and methylene groups[3,4]. The bands at 1510, 1450, 1370, and 1270 cm^{-1} are attributed to CH and NH (amide II) bending motions, skeletal vibrations of molecules, and carbon-oxygen bond vibrations[5,6]. Notably, the peak at 1510 cm-1 can be assigned to both amide II vibrations and aromatic C=C vibrations[3]. The large band at 1400 cm^{-1} may also be associated with the O–H bending vibration[7]. The band at 910 cm^{-1} is attributed to either aromatic or oleophilic C–H out-of-plane deformations[3,4]. Finally, the band at 620 cm^{-1} is likely related to ring bending associated with C=C bonds.

References:

(1) Nuzzo, A., Buurman, P., Cozzolino, V., Spacchini, R. & Piccolo, A. (2020) Infrared spectra of soil organic matter under a primary vegetation sequence. *Chemical and biological technologies in agriculture*, 7, 1-12.

(2) Kasper, M., Buchan, G. D., Mentler, A., & Blum, W. E. H. (2009). Influence of soil tillage systems on aggregate stability and the distribution of C and N in different aggregate fractions. *Soil and Tillage Research*, *105*(2), 192-199.

(3) Miralles, I., Ortega, R., Sánchez-Marañón, M., Soriano, M., & Almendros, G. (2007). Assessment of biogeochemical trends in soil organic matter sequestration in Mediterranean calcimorphic mountain soils (Almería, Southern Spain). *Soil Biology and Biochemistry*, *39*(10), 2459-2470.

(4) Coradeschi, G., Jiménez Morillo, N. T., Dias, C. B., Beltrame, M., Belo, A. D., Granged, A. J., Sadori, A. & Valera, A. (2023). Anthracological study of a Chalcolithic funerary deposit from Perdigões (Alentejo, Portugal): A new analytical methodology to establish the wood burning temperature. *Plos one*, *18*(7), e0287531.

(5) Smith, B. C. (2018). *Infrared spectral interpretation: a systematic approach*. CRC press. Boca Raton, FL, 265pp.

(6) Schindler, F. V., Mercer, E. J., & Rice, J. A. (2007). Chemical characteristics of glomalin-related soil protein (GRSP) extracted from soils of varying organic matter content. *Soil Biology and Biochemistry*, *39*(1), 320-329.

(7) MacCarthy, P. & Rice, J. A. (1985). Spectroscopic methods (other than NMR) for determining functionality in humic substances. In: *Aiken, G.R., et al. (Eds.), Humic Substances in Soil, Sediment, and Water: Geochemistry, Isolation, and Characterization*. Wiley, New York, pp. 527–559.

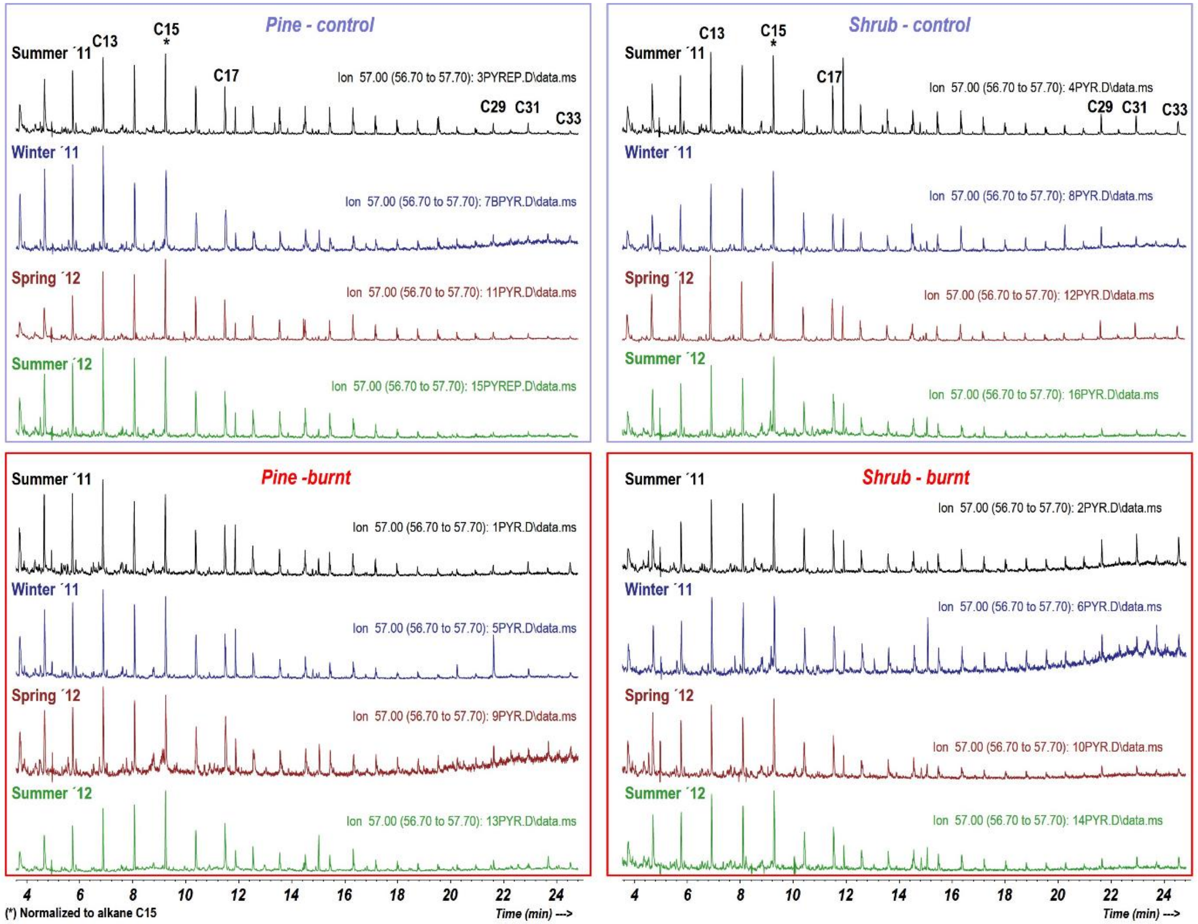

Figure S9. Single ion monitoring chromatograms for the alkane series (*m/z* 57) released after EEG pyrolysis extracted from soils under pine and shrubs, affected and not affected by fire and at different time of the year. Cn indicates the number of carbons in the alkyl chain.

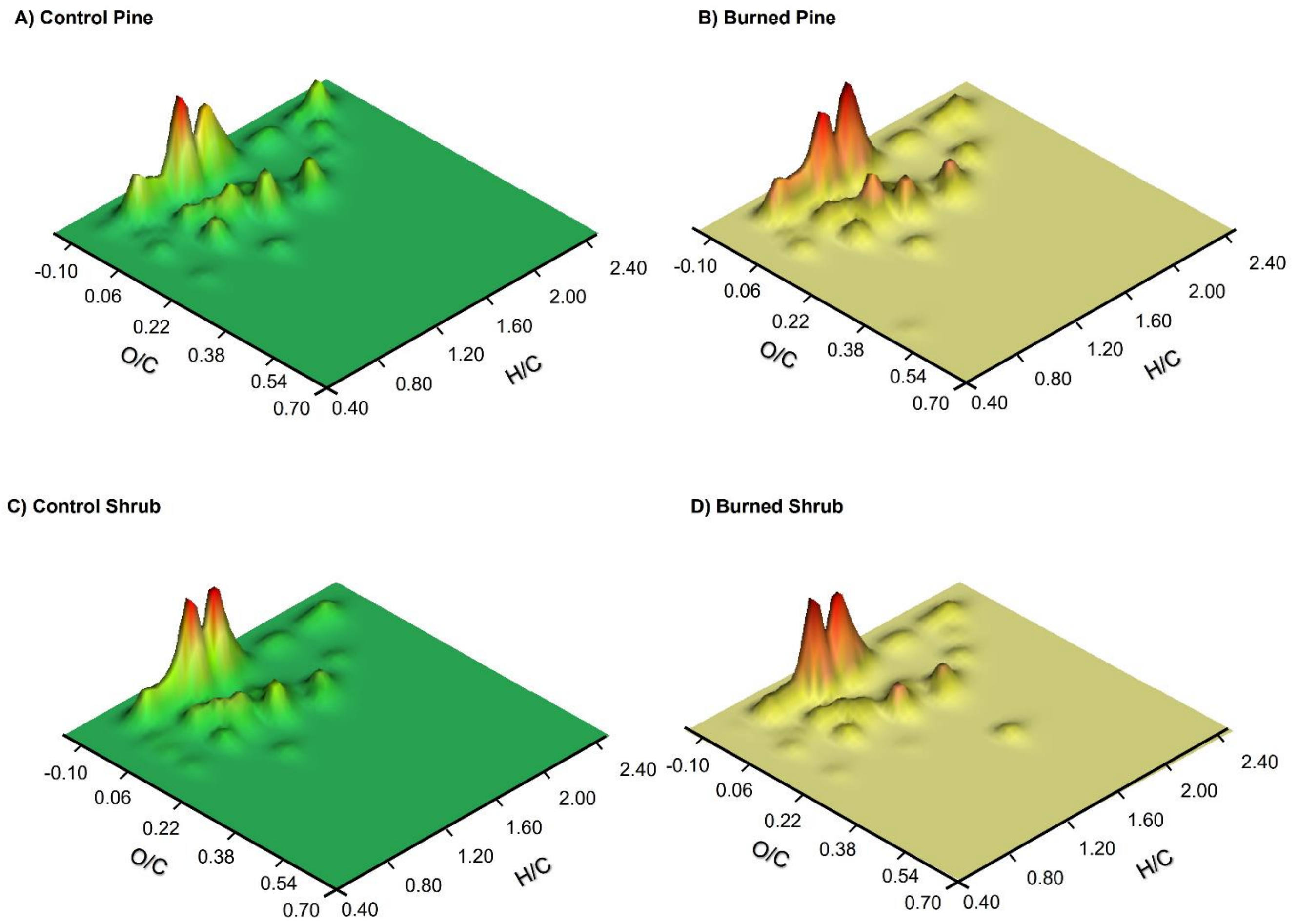

Figure S10. 3D van Krevelen diagrams that display the pyrolysis products of EEG, characterized by their H/C (x), O/C (y) atomic ratios and relative abundances (z), for a) control pine; b) burned pine; c) control shrub and d) burned shrub.

Table S2. Pyrolysis products[1] of EEG from soils affected and unaffected by forest fire, under pine and shrub coverage and different time of the year.

				PINE-BURNT				***PINE-CONTROL***				***SHRUB-BURNT***				***SHRUB-CONTROL***								
**Ref.**	**RT**	**COMPOUND**	**ORIGIN**	**Sum ´11**	**Win ´11**	**Spr ´12**	**Sum ´12**	**Sum ´11**	**Win ´11**	**Spr ´12**	**Sum ´12**	**Sum ´11**	**Win ´11**	**Spr ´12**	**Sum ´12**	**Sum ´11**	**Win ´11**	**Spr ´12**	**Sum ´12**	***MW***	***C***	***H***	***O***	***N***
1	1.67	1,3-Cyclopentadiene	LIP	6.03	8.32	9.71	6.28	7.68	8.47	0.00	6.76	8.01	11.68	5.42	5.85	3.47	8.86	6.66	6.88	66	5	6		
2	1.89	1,3-Cyclopentadiene, methyl-	LIP	6.69	9.67	13.22	11.24	9.63	10.47	13.30	10.15	13.61	15.62	10.21	11.98	6.38	14.12	9.23	11.55	80	6	8		
3	2.28	1,4,-Cyclohexadiene, 1-methyl	LIP	3.39	2.81	6.01	4.46	3.75	4.73	4.66	4.23	4.59	5.60	4.69	4.77	3.48	4.09	3.67	4.31	94	7	10		
4	2.47	BzC1 (Toluene)	ARO	4.59	5.19	7.93	3.09	3.26	8.50	4.30	4.44	4.03	5.15	4.59	3.58	3.93	4.52	4.02	3.52	92	7	8		
5	2.78	1,3-Cyclohexadiene, 5,6-dimethyl-	LIP	1.48	1.77	2.07	1.52	1.75	1.83	1.95	2.33	2.54	2.85	2.48	2.25	1.64	2.04	1.60	2.25	108	8	12		
6	2.92	Cyclopentanone	PS	2.05	2.39	2.74	1.73	2.01	2.64	1.86	1.87	1.73	1.75	1.53	1.62	1.63	1.65	2.22	1.56	84	5	8	1	
7	3.05	BzC2 (o-Xylene)	ARO	0.80	0.89	1.11	0.69	0.74	3.19	0.57	2.10	0.00	0.00	0.00	0.00	0.00	0.00	0.86	0.46	106	8	10		
8	3.12	BzC2 (m-Xylene)	ARO	1.78	2.06	2.75	1.91	1.18	0.00	1.28	0.00	2.00	2.39	2.05	1.80	1.93	1.79	1.56	1.43	106	8	10		
9	3.22	Cyclopentanone, 2-methyl-	PS	1.22	1.56	1.58	1.64	1.38	1.25	1.36	1.47	1.54	1.52	1.51	1.32	1.28	1.26	1.27	1.34	98	6	10	1	
10	3.33	BzC2 (p-Xylene)	ARO	0.99	1.13	1.36	0.97	1.04	1.44	0.97	1.02	1.01	1.30	1.14	0.99	0.69	0.98	0.97	0.97	106	8	10		
11	3.42	2-Cyclopenten-1-one	PS	3.09	1.99	0.99	2.71	3.19	3.14	2.44	3.05	3.07	3.12	2.94	2.63	3.24	2.01	3.82	2.79	98	6	10	1	
12	3.56	1H-Pyrrole, 3-methyl-	PR	0.45	0.48	3.21	0.71	0.34	0.00	0.00	0.00	0.00	0.00	0.00	0.00	0.00	0.00	0.00	0.00	81	5	7		1
13	3.60	1H-Pyrrole, 1-methyl-	PR	1.46	1.72	2.01	1.73	1.43	0.00	0.00	0.00	0.00	0.00	0.00	0.00	0.00	0.00	0.00	0.00	81	5	7		1
14	3.76	Furan, 2-ethyl	PS	0.98	1.21	1.11	0.75	1.01	1.67	2.01	1.57	1.61	1.41	1.36	1.30	1.31	1.41	1.76	1.23	96	6	8	1	
15	3.86	BzC3 (Benzene, -ethyl-methyl-)	ARO	0.53	0.67	0.78	0.46	0.48	0.73	0.75	0.00	0.80	0.86	0.82	0.82	0.80	0.82	1.36	0.68	120	9	12		
16	3.90	2-Cyclopenten-1-one, 2-methyl-	PS	2.60	2.82	3.87	3.25	2.64	3.55	3.23	3.38	3.13	3.50	3.46	3.04	2.98	2.88	3.35	3.11	96	6	8	1	
17	4.06	Cyclohexene, 1,2-dimethyl-	LIP	1.64	1.50	2.11	1.61	1.59	1.42	1.35	1.56	1.40	1.45	1.54	1.35	1.30	1.22	1.36	1.31	110	8	14		
18	4.22	BzC3 (Benzene, -trimethyl)	ARO	1.25	1.32	2.03	1.42	1.45	2.08	1.30	1.58	1.49	1.58	1.54	1.41	1.48	1.36	2.07	1.28	120	6	12		
19	4.35	BzC2:1 (Benzene, -propenyl-)	ARO	0.56	0.70	0.74	0.92	1.51	0.83	1.44	1.46	1.48	1.44	1.38	1.25	1.26	1.27	1.34	1.19	117	9	10		
20	4.54	Benzofuran	PS	1.10	1.26	1.83	1.11	0.95	1.42	0.81	1.16	0.72	0.95	1.03	0.84	0.93	0.68	1.19	0.80	118	8	6	1	
21	4.69	BzC2:1 (Benzene, -ethenyl-methyl-)	ARO	0.78	0.92	0.91	0.78	0.73	1.19	0.58	0.81	0.58	0.99	0.60	0.52	0.80	0.50	1.14	0.71	117	9	10		
22	4.77	2-Cyclopenten-1-one, 2,3-dimethyl-	PS	1.14	1.19	0.83	0.73	1.04	0.83	0.78	0.98	0.89	0.69	0.96	0.88	0.74	0.80	0.81	0.66	110	7	10	1	
23	4.84	2,4-Dimethylfuran	PS	1.06	1.51	1.36	1.27	1.47	0.75	1.36	1.27	1.30	0.63	1.00	1.08	1.29	1.30	0.97	1.02	96	6	8	1	
24	4.96	Indene	ARO	1.40	1.49	1.75	1.50	1.29	2.51	1.88	2.20	1.98	2.04	2.23	1.98	1.67	2.05	1.74	1.75	116	9	8		
25	5.19	3-Acetylpyrrole	PR	0.69	0.76	0.00	0.80	0.61	0.00	0.91	0.00	0.78	0.00	0.56	0.79	0.88	0.70	0.76	0.79	109	6	7	1	1
26	5.44	Isomer of 22	PS	1.43	1.22	1.27	1.23	1.21	0.30	2.16	1.57	2.25	1.09	2.65	1.39	2.70	2.08	1.82	2.53	110	7	10	1	
27	5.51	2-Cyclopenten-1-one, 2,3,4-trimethyl-	PS	0.70	0.94	0.69	0.53	0.71	0.91	0.00	0.82	0.00	0.61	0.00	0.58	0.00	0.54	0.00	0.00	124	8	12	1	

28	5.58	Benzofuran, 2-methyl-	PS	0.45	0.59	0.59	0.34	0.41	0.80	0.00	0.61	0.00	0.52	0.00	0.51	0.00	0.00	0.41	0.00	131	9	8	1	
29	5.67	Phenol	ARO	3.83	3.93	4.83	2.80	3.01	3.96	2.57	3.42	2.16	2.37	2.81	2.48	1.90	2.10	3.16	2.43	94	6	6	1	
30	5.68	Isomer of 27	PS	0.00	0.00	0.00	0.00	0.00	0.00	0.00	0.00	0.00	0.44	0.45	0.00	1.34	0.00	0.00	0.00	124	8	12	1	
31	5.98	Phenol, 2-methoxy-	LIG	1.79	1.55	0.00	1.42	1.91	0.37	1.37	1.28	1.05	0.28	0.00	0.80	1.39	0.91	0.84	0.88	124	7	8	2	
32	6.10	1H-Indene, 1-methyl-	ARO	1.41	2.01	1.69	1.72	1.36	1.39	1.90	1.75	1.85	1.40	1.92	1.82	1.81	1.85	1.42	2.00	130	10	10		
33	6.27	PhC1 (o-Cresol)	ARO	1.68	1.70	0.72	2.04	1.60	0.73	1.62	1.29	2.24	0.00	2.79	1.48	1.62	2.30	1.56	2.06	108	7	8	1	
34	6.34	PhC1 (m-Cresol)	ARO	0.50	0.53	0.00	0.00	0.49	0.00	0.84	0.99	0.00	1.11	0.00	0.83	0.73	0.00	0.00	0.00	108	7	8	1	
35	6.35	PhC1 (p-Cresol)	ARO	0.44	0.50	0.81	0.00	0.00	0.00	0.00	0.00	0.00	1.07	0.00	0.00	0.00	0.00	0.00	0.00	108	7	8	1	
36	6.47	PhC2 (Phenol, -dimethyl-)	ARO	0.00	0.00	0.00	0.41	0.28	0.00	0.00	0.00	0.00	0.00	0.00	0.00	0.00	0.00	0.00	0.00	122	8	10	1	
37	6.55	Cyclohexane, 1-methyl-4-(1-methylethylidene)-	TER	1.37	0.85	0.42	0.83	0.94	0.71	1.12	1.30	1.07	1.01	1.21	1.09	1.25	0.95	0.87	1.16	138	10	18		
38	6.71	PhC2 (Phenol, -dimethyl-)	ARO	0.88	0.00	0.70	1.47	0.61	0.00	1.51	0.00	0.00	0.00	0.49	0.41	1.84	0.53	0.47	0.41	122	8	10	1	
39	6.73	PhC2 (Phenol, -dimethyl-)	ARO	1.38	1.81	0.00	0.31	1.29	0.00	0.00	1.55	0.77	1.64	0.73	0.85	0.00	0.78	0.71	0.75	122	8	10	1	
40	6.83	Azulene	HAR	0.45	0.39	0.68	0.22	0.00	0.00	0.00	0.00	0.00	0.00	1.44	0.40	0.60	0.36	0.00	0.48	128	10	8		
41	6.88	Naphthalene	PAH	0.40	0.67	0.71	0.43	0.00	0.00	0.60	0.00	0.00	0.00	0.00	0.62	0.00	0.00	0.00	0.00	128	10	8		
42	6.91	Alkene C13	LIP	0.56	0.90	0.71	0.43	0.59	2.14	0.37	1.09	0.99	0.00	0.57	0.00	0.49	0.00	1.67	0.68	184	13	28		
43	7.00	Ethanone, 1-(2-methylphenyl)-	LIG	0.43	0.27	0.37	0.59	0.34	0.55	0.74	0.00	0.64	0.38	0.00	0.77	0.55	0.59	0.65	0.61	134	9	10	1	
44	7.11	Benzonitrile, 2-methyl-	ARO	0.44	0.41	0.20	0.25	0.33	0.00	0.00	0.00	0.00	0.00	0.00	0.00	0.26	0.00	0.28	0.00	117	8	7		1
45	7.14	Benzene, (2-methyl-1-butenyl)-	ARO	0.00	0.00	0.00	0.00	0.00	0.00	0.00	0.00	0.00	0.00	0.27	0.00	0.96	0.00	0.61	0.00	146	11	14		
46	7.21	PhC2 (Phenol,-ethyl-)	ARO	1.06	0.77	0.20	0.51	1.05	0.00	1.37	0.00	0.77	0.00	0.00	0.69	0.00	0.71	0.00	0.90	122	8	10	1	
47	7.25	Isomer of 43	LIG	0.00	0.00	0.00	0.00	0.00	0.00	0.00	0.00	0.00	0.00	0.42	0.00	0.00	0.00	0.00	0.00	134	9	10	1	
48	7.31	PhC2 (Phenol, -dimethyl-)	ARO	1.08	0.82	0.00	1.04	2.06	0.00	1.80	0.74	0.00	0.00	0.00	0.00	1.72	0.00	0.00	0.00	122	8	10	1	
49	7.35	1H-Indene, 1,3-dimethyl-	HAR	0.72	0.92	0.30	0.94	0.00	0.32	0.63	1.45	2.05	1.15	1.38	1.55	0.00	1.61	1.22	1.41	144	11	12		
50	7.50	Naphthalene, 1,2-dihydro-3-methyl-	HAR	0.52	0.71	0.49	0.66	0.49	0.30	0.00	0.00	0.00	0.61	0.70	0.67	0.54	0.60	0.00	0.63	144	11	12		
51	7.60	Benzaldehyde, 2,4-dimethyl-	LIG	1.17	2.57	0.25	1.55	1.32	9.00	1.24	2.58	3.06	2.76	0.00	2.79	3.26	2.66	10.49	2.86	134	9	10	1	
52	7.63	Isomer of 49	HAR	1.51	0.22	1.95	0.90	1.51	0.00	0.96	0.00	0.00	0.00	2.75	0.00	0.00	0.00	0.00	0.00	144	11	12		
53	7.79	PhC2 (Phenol,-ethyl-)	ARO	1.96	1.67	0.38	1.90	2.03	0.84	1.45	1.29	1.26	0.00	0.00	1.02	1.59	1.05	1.46	0.94	122	8	10	1	
54	7.86	1H-Indene, 1-ethylidene-	HAR	0.00	0.00	0.00	0.00	0.00	0.00	0.00	0.00	0.00	0.37	0.00	0.00	0.00	0.00	0.00	0.00	142	11	10		
55	7.96	1,2,3-Trimethylindene	HAR	0.64	0.69	0.76	0.36	0.37	0.66	0.70	1.15	0.66	1.11	1.34	0.80	0.00	0.73	0.00	0.67	158	12	14		
56	7.99	b-Damascone (177, 192)	TER	0.00	0.00	0.00	0.00	0.60	0.00	0.00	0.00	0.00	0.00	0.00	0.00	1.25	0.00	0.00	0.00	192	13	20	1	
57	8.04	Benzocycloheptatriene	HAR	0.33	0.00	0.00	0.31	0.00	0.00	0.00	0.00	0.00	0.00	0.00	0.00	0.00	0.00	0.00	0.42	141	11	10		
58	8.11	Alkane C14	LIP	0.52	0.63	0.30	0.70	0.50	0.80	0.45	0.79	0.36	0.48	0.54	0.38	0.00	0.61	0.97	0.43	198	14	30		
59	8.16	.alpha.-Terpinen	TER	0.49	0.40	0.00	0.00	1.05	0.00	0.00	0.00	0.00	0.00	0.00	0.00	0.00	0.00	0.00	0.00	136	10	16		
60	8.22	NaphC1 (Naphthalene, -methyl-)	PAH	0.43	0.93	0.49	0.95	0.66	0.82	1.37	1.09	1.06	0.57	0.98	1.12	1.87	1.04	0.97	1.09	142	11	10		

61	8.36	PhC3 (Phenol,-ethyl--methyl-)	ARO	0.72	0.62	0.26	0.71	1.16	0.00	1.05	0.51	0.62	0.00	0.00	0.00	2.02	0.84	0.00	0.87	136	9	12	1	
62	8.42	1-Naphthalenol	PAH	0.00	0.00	0.00	0.00	0.00	0.00	0.00	0.00	0.00	0.26	0.44	0.00	0.00	0.00	0.00	0.00	144	10	8	1	
63	8.42	NaphC1 (Naphthalene, -methyl-)	PAH	0.37	0.37	0.35	0.53	0.00	0.00	0.00	0.47	0.00	0.00	0.49	0.91	0.00	0.00	0.82	0.00	142	11	10		
64	8.43	2,5-Pyrrolidinedione, 3-ethyl-4-methyl-	PR	0.00	0.00	0.00	0.00	0.00	0.00	0.00	0.00	1.63	0.00	0.00	0.00	0.00	0.00	0.00	0.00	141	7	11	2	1
65	8.48	PhC3 (Phenol,-trimethyl-	ARO	0.66	0.58	0.00	0.42	0.61	0.75	0.00	0.64	0.00	0.00	0.00	0.00	0.00	0.00	0.00	0.00	136	9	12	1	
66	8.48	5,6-Dihydro-6-methyluracil	PR	0.00	0.00	0.00	0.00	0.00	0.00	0.00	0.00	0.00	1.79	0.00	0.00	0.00	0.00	0.00	0.00	128	5	8	2	2
67	8.54	(1-Methylpenta-2,4-dienyl)benzene	ARO	0.00	0.00	0.00	0.00	0.00	0.00	0.00	0.00	0.00	0.00	0.51	0.54	1.36	0.00	0.00	0.50	158	12	14		
68	8.57	PhC3 (Phenol,-trimethyl-	ARO	0.26	0.00	0.57	0.25	0.32	0.42	0.00	0.00	0.00	0.00	0.36	0.00	0.00	0.00	0.00	0.00	136	9	12	1	
69	8.71	2-Methyl-1-indanone	HAR	0.82	0.65	0.00	0.63	0.81	0.81	1.43	0.63	1.32	0.38	0.58	1.42	0.00	1.08	1.62	1.32	146	10	10	1	
70	8.76	Isomer of 69	HAR	0.36	0.88	0.60	0.41	0.28	0.63	0.66	0.80	0.00	1.12	0.71	0.00	0.46	0.00	0.00	0.00	146	10	10	1	
71	8.83	1H-indene, 1,1,3-trimethyl-	HAR	0.41	0.00	0.00	0.64	0.66	0.00	0.00	0.00	0.00	0.00	0.00	0.00	0.00	0.00	0.00	0.00	158	12	14		
72	8.92	Isomer of 71	HAR	0.60	0.34	0.15	0.32	0.00	0.00	0.00	0.50	1.15	0.65	0.54	1.03	0.56	0.86	0.00	0.84	158	12	14		
73	8.97	Isomer of 69	HAR	0.30	0.31	0.00	0.57	1.51	0.51	1.07	0.00	0.00	0.00	0.00	0.00	0.00	0.00	0.00	0.00	146	10	10	1	
74	9.03	Isomer of 71	HAR	1.00	0.78	0.24	0.00	0.00	0.00	0.00	0.83	0.78	0.00	0.33	0.64	0.00	0.63	0.51	0.67	158	12	14		
75	9.08	Isomer of 69	HAR	0.00	0.00	0.00	0.00	0.00	0.00	0.00	0.00	0.00	0.48	0.57	0.00	1.11	0.00	0.00	0.00	146	10	10	1	
76	9.15	Phenol, 5-methyl-2-(1-methylethyl)- (Thymol)	TER	0.50	0.43	0.00	0.58	0.61	0.00	0.74	0.78	0.59	0.00	0.00	0.67	1.01	0.58	0.00	0.74	150	10	14	1	
77	9.30	Alkane C15	LIP	1.06	0.66	0.30	0.75	1.18	1.50	1.10	1.78	0.83	0.45	1.41	0.78	0.81	0.84	1.41	1.31	212	15	32		
78	9.40	Naphthalene, 1-(methoxymethyl)-	LIG	0.00	0.00	0.00	0.00	0.00	0.00	0.00	0.00	0.00	0.00	0.00	0.45	0.65	0.41	0.00	0.00	172	12	12	1	
79	9.60	NaphC2 (Naphthalene, -dimethyl-)	PAH	0.81	0.80	0.24	0.44	0.00	0.00	0.00	0.00	0.50	0.00	0.58	1.13	1.36	0.57	0.00	0.69	156	12	12		
80	9.66	Indol	PR	0.54	0.47	0.35	0.60	0.66	0.00	1.24	1.15	0.54	0.00	0.43	0.00	0.00	0.44	0.85	0.43	117	8	7		1
81	9.74	NaphC2 (Naphthalene, -dimethyl-)	PAH	0.30	0.28	0.21	0.38	0.29	0.48	0.43	0.39	0.30	0.59	0.00	0.00	0.00	0.00	0.00	0.00	156	12	12		
82	9.79	NaphC2 (Naphthalene, -dimethyl-)	PAH	0.41	0.49	0.25	0.43	0.83	0.00	0.45	0.69	0.40	0.48	0.39	0.34	1.12	0.29	0.00	0.35	156	12	12		
83	9.85	NaphC2 (Naphthalene, -dimethyl-)	PAH	0.00	0.00	0.00	0.00	0.00	0.00	0.00	0.00	0.00	0.39	0.62	0.57	0.00	0.31	0.00	0.55	156	12	12		
84	9.95	Naphthalene, 1,2-dihydro-3,5,8-trimethyl-	HAR	0.00	0.00	0.00	0.00	0.00	0.00	0.00	0.00	0.00	0.00	0.33	0.37	0.51	0.38	0.26	0.35	172	13	16		
85	10.07	1H-Indene-4-carboxaldehyde, 2,3-dihydro-	HAR	0.47	0.56	0.00	0.78	0.71	0.00	1.66	0.00	1.16	1.14	1.65	1.60	1.47	1.36	1.14	1.70	146	10	10	1	
86	10.14	Isomer of 85	HAR	0.00	0.00	0.59	0.23	0.56	1.07	0.00	1.51	0.00	0.00	0.00	0.00	0.00	0.00	0.00	0.00	146	10	10	1	
87	10.34	Benzene, 1-(1-methylethenyl)-2-(1-methylethyl)-	ARO	0.60	0.69	0.00	0.44	0.00	0.00	1.06	0.00	0.42	0.00	0.86	0.50	1.05	0.47	0.78	0.91	160	12	16		
88	10.45	AlkaneC16	LIP	1.24	0.52	0.12	0.49	0.00	0.96	0.00	0.00	0.00	0.72	0.00	0.00	1.22	0.00	1.12	0.00	226	16	34		
89	10.43	Benzene, (1-ethyl-1-propenyl)-	ARO	0.00	0.00	0.00	0.00	0.90	0.00	0.00	0.00	0.85	0.00	0.00	1.10	0.00	0.82	0.00	1.20	146	11	14		
90	10.55	1H-Inden-1-one, 2,3-dihydro-3,3-dimethyl-	HAR	0.00	0.00	0.00	0.00	0.37	0.00	0.81	0.00	0.28	0.00	0.00	0.00	1.25	0.00	0.00	0.00	160	11	12	1	
91	10.55	1(3H)-Isobenzofuranone	ARO	0.00	0.00	0.28	0.00	0.00	0.57	0.00	0.57	0.00	0.44	1.57	0.00	0.33	0.00	0.00	0.00	134	8	6	2	
92	10.91	9-Octadecen-1-ol (Z)	LIP	0.00	0.00	0.00	0.00	0.00	0.49	0.00	0.82	0.00	0.52	0.67	0.00	0.00	0.00	0.00	0.41	268	18	36	1	

93	10.94	NaphC3 (Naphthalene, x,x,x-trimethyl-)	PAH	0.00	0.00	0.00	0.00	0.00	0.00	1.21	0.00	0.61	0.00	0.47	0.48	0.51	0.39	0.00	0.99	170	13	14		
94	10.99	Isomer of 90	HAR	0.60	0.37	0.00	0.48	0.51	0.00	0.00	0.00	0.00	0.00	0.00	0.00	0.00	0.00	0.00	0.00	160	11	12	1	
95	11.08	NaphC3 (Naphthalene, x,x,x-trimethyl-)	PAH	0.00	0.00	0.00	0.00	0.00	0.00	0.00	0.00	0.37	0.21	0.51	0.00	0.00	0.42	0.00	0.60	170	13	14		
96	11.24	Trimethylazulene	HAR	0.00	0.00	0.00	0.00	0.00	0.00	0.00	0.00	0.28	0.00	0.67	0.62	0.00	0.33	0.00	0.00	170	13	14		
97	11.46	Isomer of 96	HAR	0.00	0.00	0.00	0.00	0.00	0.00	0.00	0.00	0.43	0.00	0.59	0.53	0.00	0.69	0.00	0.78	170	13	14		
98	11.47	Chamazulene	HAR	0.77	0.33	0.00	0.33	0.67	0.00	0.00	0.00	0.00	0.00	0.00	0.00	0.00	0.00	0.00	0.00	184	14	16		
99	11.56	Alkane C17	LIP	0.95	0.64	0.64	0.62	0.88	0.60	0.78	0.99	0.57	0.65	0.63	0.65	0.81	0.61	1.44	0.72	240	17	36		
100	11.80	NaphC3 (Naphthalene, x,x,x-trimethyl-)	PAH	0.90	0.78	0.75	0.63	0.95	0.00	0.00	0.00	0.00	0.00	0.00	0.00	0.00	0.00	0.00	0.00	170	13	14		
101	11.93	cis-2-Methyl-7-octadecene	LIP	0.57	0.47	0.48	0.52	0.40	0.69	0.00	0.40	0.00	0.00	0.00	0.00	1.01	0.00	1.65	0.00	266	19	38		
102	11.98	9H-Fluorene	PAH	0.39	0.29	0.00	0.38	0.34	0.00	1.87	0.37	0.59	0.00	1.87	0.67	0.00	0.64	0.00	0.74	166	13	10		
103	12.02	9H-Fluorene, 2-methyl-	PAH	0.44	0.44	0.70	0.70	0.81	1.00	0.00	1.36	0.75	1.03	0.63	1.01	1.04	0.84	0.00	1.08	180	14	12		
104	12.60	Alkane C18	LIP	0.00	0.00	0.00	0.00	0.00	0.00	0.00	0.00	0.00	0.00	0.00	0.00	0.60	0.00	0.47	0.00	254	18	38		
105	12.77	Anthracene, -octahydro-	HAR	0.00	0.00	0.00	0.00	0.00	0.00	0.00	0.00	0.00	0.00	0.00	0.00	1.04	0.00	0.00	0.00	186	14	18		
106	12.83	2-Hidroxyfluorene	PAH	0.64	0.25	0.00	0.67	0.00	0.15	0.78	0.81	0.55	0.21	0.70	0.88	0.00	0.71	0.00	1.15	182	13	10	1	
107	13.07	Hexadecenoic acid	LIP	0.32	0.00	0.14	0.69	0.00	0.19	0.00	0.21	0.35	0.22	0.00	0.41	0.00	0.43	0.47	0.54	254	16	30	2	
108	13.13	Isomer of 103	PAH	0.00	0.00	0.00	0.36	0.50	0.00	0.44	0.82	0.28	0.34	0.51	0.76	1.03	0.72	0.40	0.81	180	14	12		
109	13.21	Phenanthrene, 9,10-dihydro-	HAR	0.42	0.32	0.00	0.53	0.00	0.00	0.67	0.00	0.26	0.00	0.00	0.00	0.00	0.00	0.00	0.00	180	14	12		
110	13.27	Isomer of 103	PAH	0.25	0.13	0.00	0.33	1.38	0.00	0.92	0.65	0.00	0.37	0.27	0.88	0.00	0.61	0.46	0.73	180	14	12		
111	13.42	3H-Benz[e]indene, 2-methyl-	HAR	0.60	0.28	0.00	0.46	0.00	0.24	0.00	0.00	0.50	0.00	0.77	0.00	0.00	0.00	0.00	0.00	180	14	12		
112	13.62	Alkane C19	LIP	0.57	0.10	0.00	0.00	0.20	0.00	0.00	0.68	0.00	0.00	0.00	0.00	1.32	0.00	0.43	0.00	268	19	40		
113	13.78	1-Naphthalenol, 3-methyl-	PAH	0.42	0.00	0.00	0.50	0.50	0.00	1.01	0.00	0.00	0.00	0.18	0.88	1.06	0.51	0.00	0.00	158	11	10	1	
114	13.82	1-Naphthol, 6,7-dimethyl-	PAH	0.00	0.00	0.00	0.45	0.00	0.00	0.81	0.00	0.28	0.00	0.00	0.00	0.54	0.58	0.00	0.00	172	12	12	1	
115	13.94	Phenanthrene	PAH	0.00	0.00	0.00	0.00	0.00	0.00	0.00	0.61	0.00	0.16	0.52	0.56	0.00	0.00	0.00	0.56	178	14	10		
116	13.95	2-Pentadecanone, 6,10,14-trimethyl-	LIP	0.00	0.00	0.00	0.00	0.00	0.00	0.00	0.00	0.00	0.00	0.00	0.00	0.00	0.00	0.23	0.00	268	18	36	1	
117	14.02	9H-Fluorene, 9-propyl-	PAH	0.19	0.00	0.00	0.00	0.00	0.00	0.66	0.00	0.00	0.00	0.00	0.55	0.54	0.00	0.00	0.00	208	16	16		
118	14.07	Benzofuran-2-one, 2,3-dihydro-4-nitro-	PR	0.14	0.03	0.00	0.28	0.00	0.00	0.48	0.00	0.00	0.00	0.41	0.00	0.00	0.00	0.00	0.44	179	8	5	4	1
119	14.29	9-Methoxy-fluorene	PAH	0.00	0.00	0.00	0.00	0.00	0.00	0.00	0.00	0.00	0.00	0.35	0.43	0.00	0.25	0.00	0.40	196	14	12	1	
120	14.32	Anthracene, 9,10-dihydro-2-methyl-	HAR	0.00	0.00	0.00	0.00	0.00	0.00	0.00	0.00	0.36	0.00	0.00	0.00	0.00	0.00	0.00	0.00	194	15	14		
121	14.50	Hexadecanoic acid, methyl ester	LIP	0.45	0.13	0.00	0.29	0.36	0.20	0.49	1.13	0.00	0.12	0.00	0.49	0.00	0.00	0.53	0.00	270	17	34	1	
122	14.55	Isomer of 120	HAR	0.00	0.00	0.00	0.00	0.00	0.00	0.00	0.00	0.24	0.00	0.00	0.49	0.54	0.34	0.00	0.32	194	15	14		
123	14.58	Alkane C20	LIP	0.19	0.20	0.34	0.34	0.51	0.00	0.00	0.00	0.00	0.55	0.00	0.00	0.61	0.67	0.62	0.52	282	20	42		
124	14.59	Isomer of 120	LIP	0.00	0.00	0.00	0.00	0.00	0.00	0.00	0.00	0.15	0.00	0.72	0.67	0.00	0.00	0.00	0.75	194	15	14		
125	14.64	4-Hydroxy-1-naphthaldehyde	PAH	0.00	0.00	0.00	0.00	0.00	0.28	0.51	0.00	0.00	0.00	0.00	0.00	0.00	0.69	0.00	0.00	172	11	8	2	

126	14.76	Isomer of 120	HAR	0.00	0.00	0.00	0.00	0.00	0.00	0.00	0.00	0.42	0.00	0.66	0.00	0.00	0.00	0.00	0.00	194	15	14	
127	14.97	Isomer of 120	HAR	0.00	0.00	0.00	0.00	0.00	0.00	0.53	0.00	0.00	0.00	0.00	0.00	0.00	0.19	0.00	0.00	194	15	14	
128	15.10	Palmitoleic acid	LIP	0.58	0.23	0.58	1.24	0.33	0.79	0.48	0.69	0.39	1.14	0.00	0.51	0.48	0.52	0.63	0.56	268	17	32	2
129	15.26	1-Naphthol, 2,5,8-trimethyl-	PAH	0.00	0.00	0.00	0.00	0.00	0.00	0.57	0.00	0.00	0.00	0.00	0.00	0.00	0.26	0.00	0.00	186	13	14	1
130	15.51	2-Hydroxy-4-isopropylnaphthalene	PAH	0.00	0.00	0.00	0.00	0.00	0.00	0.00	0.00	0.00	0.00	0.00	0.00	0.00	0.45	0.00	0.00	186	13	14	1
131	15.53	Alkane C21	LIP	0.00	0.00	0.00	0.00	0.00	0.00	0.00	0.00	0.25	0.00	0.00	0.47	0.69	0.00	0.21	0.00	296	21	44	
132	15.75	Anthrone	HAR	0.00	0.00	0.00	0.00	0.00	0.00	0.00	0.00	0.00	0.00	0.00	0.21	0.00	0.25	0.00	0.00	194	14	10	1
133	15.97	Phenanthrene, 1-methyl-	PAH	0.27	0.06	0.00	0.37	0.00	0.00	0.44	0.33	0.00	0.00	0.00	0.32	0.00	0.00	0.00	0.00	192	15	12	
134	16.39	Octadecanoic acid, methyl ester	LIP	0.00	0.00	0.00	0.34	0.17	0.08	1.05	0.36	0.14	0.00	0.00	0.13	0.32	0.00	0.16	0.25	298	19	38	2
135	16.40	Alkane C22	LIP	0.00	0.00	0.00	0.00	0.00	0.00	0.00	0.00	0.00	0.00	0.00	0.00	0.34	0.22	0.27	0.19	310	22	46	
136	16.75	10,18-Bisnorabieta-5,7,9(10),11,13-pentaene	LIP	0.58	0.28	0.00	0.33	0.28	0.00	0.33	0.00	0.12	0.00	0.00	0.00	0.00	0.17	0.00	0.00	238	18	22	
137	16.92	Octadecenoic acid	LIP	0.00	0.00	0.00	0.46	0.00	0.00	0.00	0.00	0.00	0.09	0.00	0.15	0.00	0.00	0.06	0.00	282	18	34	2
138	17.12	Phenanthrene, 3,6-dimethyl-	PAH	0.48	0.25	0.00	0.49	0.34	0.00	0.00	0.14	0.00	0.00	0.00	0.00	0.00	0.00	0.00	0.00	206	16	14	
139	18.28	Retene	PAH	1.55	1.06	0.36	0.53	0.54	0.36	0.42	0.23	0.21	0.00	0.07	0.12	0.48	0.33	0.18	0.07	234	18	18	

[1]Calculated as percentage of total ion chromatographic area. [2]PS: polysaccharides, PR: proteins and polypeptides, LIP: lipids, PAH: polycyclic aromatic hydrocarbons, HAR: hydro-aromatics, ARO: non-specific aromatic compounds, LIG: lignin and polyphenols, TER: terpenes. Sum: Summer; Wint: Winter; Spri: Spring. Molecular weight (MW) and elemental composition are also included.
